# Ensemble machine learning prediction and variable importance analysis of 5-year mortality after cardiac valve and CABG operations

**DOI:** 10.1038/s41598-021-82403-0

**Published:** 2021-02-10

**Authors:** José Castela Forte, Hubert E. Mungroop, Fred de Geus, Maureen L. van der Grinten, Hjalmar R. Bouma, Ville Pettilä, Thomas W. L. Scheeren, Maarten W. N. Nijsten, Massimo A. Mariani, Iwan C. C. van der Horst, Robert H. Henning, Marco A. Wiering, Anne H. Epema

**Affiliations:** 1 Department of Clinical Pharmacy and Pharmacology, University of Groningen, University Medical Center Groningen, Hanzeplein 1, P.O. Box 30.001, 9700 RB Groningen, The Netherlands; 2 Department of Anesthesiology, University of Groningen, University Medical Center Groningen, Groningen, The Netherlands; 3 Department of Internal Medicine, University of Groningen, University Medical Center Groningen, Groningen, The Netherlands; 4 Division of Intensive Care Medicine, Department of Anesthesiology, Intensive Care and Pain Medicine, University of Helsinki and Helsinki University Hospital, Helsinki, Finland; 5 Department of Critical Care, University of Groningen, University Medical Center Groningen, Groningen, The Netherlands; 6 Department of Cardiothoracic Surgery, University of Groningen, University Medical Center Groningen, Groningen, The Netherlands; 7 Bernoulli Institute for Mathematics, Computer Science and Artificial Intelligence, University of Groningen, Groningen, The Netherlands; 8 Department of Intensive Care, Maastricht University Medical Centre+, University Maastricht, Maastricht, The Netherlands

**Keywords:** Biomarkers, Medical research, Risk factors, Mathematics and computing

## Abstract

Despite having a similar post-operative complication profile, cardiac valve operations are associated with a higher mortality rate compared to coronary artery bypass grafting (CABG) operations. For long-term mortality, few predictors are known. In this study, we applied an ensemble machine learning (ML) algorithm to 88 routinely collected peri-operative variables to predict 5-year mortality after different types of cardiac operations. The Super Learner algorithm was trained using prospectively collected peri-operative data from 8241 patients who underwent cardiac valve, CABG and combined operations. Model performance and calibration were determined for all models, and variable importance analysis was conducted for all peri-operative parameters. Results showed that the predictive accuracy was the highest for solitary mitral (0.846 [95% CI 0.812–0.880]) and solitary aortic (0.838 [0.813–0.864]) valve operations, confirming that ensemble ML using routine data collected perioperatively can predict 5-year mortality after cardiac operations with high accuracy. Additionally, post-operative urea was identified as a novel and strong predictor of mortality for several types of operation, having a seemingly additive effect to better known risk factors such as age and postoperative creatinine.

## Introduction

Whereas complications after cardiac operations are associated with increased risk of in-hospital mortality, only few predict long-term mortality. The best documented is post-operative acute kidney injury (AKI), a highly prevalent complication occurring in 15–30% of patients^1,2^ which is associated with both increased short- and long-term mortality^1–4^. The relation between postoperative AKI and mortality varies greatly per type of cardiac operation. Mortality risks related to AKI are well characterized for coronary artery bypass grafting (CABG), but less well studied in valve operations, despite these accounting for 24% of all cardiac operations and having higher mortality rates^5,6^. Recently, Bouma et al.^5^, showed post-operative AKI to be strongly associated with an increase in long-term mortality in patients with solitary valve and combined valve and CABG operations. Remarkably, even a mild impairment in renal function well below the threshold for AKI-1 (i.e., a mere 10% post-operative increase in serum creatinine) significantly increased long-term mortality risk in solitary valve operations^5^. Therefore, to date postoperative AKI represents the best studied organ injury related early marker of long-term mortality risk after cardiac operations.

Previously, we have demonstrated that machine learning (ML) predictive models proved superior to classical multivariable analysis in identifying patients at increased risk of long-term mortality after CABG operations^7^. Moreover, a unique property of ML is its ability to identify parameters predicting mortality and rank their importance by variable importance analysis. However, while ML analyses gain popularity in peri-operative care^8^, studies using ML techniques for long-term mortality analysis after cardiac valve operations are lacking. Several studies in different fields of healthcare have shown ensemble ML algorithms to be more accurate than individual algorithms in modelling complex outcomes such as mortality in critically ill patients^9^ and mortality following cardiac arrest^10^. In anesthesiology, recent studies showed that different machine learning algorithms could accurately predict acute hypotensive episodes 10 min in advance using patient characteristics and physiological variables^11–13^.

In this study, we combined multiple ML algorithms into an ensemble using the Super Learner (SL) algorithm^14^. This ensemble ML algorithm was trained to predict 5-year mortality in a large prospective cohort of patients undergoing cardiac valve, CABG, or combined operations using routinely collected peri-operative data in a single tertiary care hospital. We compared the accuracy of two SL training methodologies, using a targeted approach with patients split per operation type compared to the entire, unselected population. Furthermore, variable importance analysis was conducted to identify the strongest predictors of mortality.

## Results

### Patient characteristics and mortality per operation type

Patient characteristics, descriptives of all variables used in this study and mortality data per operation type are summarized in Table 1 (and Table 1 of the “Supplementary material”). Five years mortality rate of the full patient cohort was 16.5%. Operations involving valve procedures showed higher mortality amounting 16.9% for aortic valve alone, 19.7% for mitral valve alone, 21.0% for combined aortic valve/CABG and 28.9% for combined mitral valve/CABG (Table 1). Accordingly, mortality rate for CABG-only (13.8%) was lower than for the entire cohort.

**Table 1:** Descriptives table per operation type.

	CABG	Aortic valve	Mitral valve	Aortic + coronary	Mitral + coronary	P value
	N = 4514	N = 1663	N = 884	N = 813	N = 367	
BMI	27.34	27.01	26.03	27.40	26.56	< 0.001
Gender						< 0.001
Female	1028 (22.77%)	676 (40.65%)	424 (47.96%)	285 (35.06%)	125 (34.06%)	
Male	3486 (77.23%)	987 (59.35%)	460 (52.04%)	528 (64.94%)	242 (65.94%)	
Age	66.26	64.80 (13.79)	62.62 (13.62)	72.25 (8.39)	69.28 (8.57)	< 0.001
Pre-operative eCCR	71.42	72.32 (21.84)	74.83	74.54	65.78	0.065
Post-operative eCCR	66.99	70.50 (39.87)	67.43 (27.26)	62.94 (24.22)	59.03 (23.90)	< 0.001
Per-operative eCCR decrease	4.44	1.82 (32.85)	7.40 (95.76)	11.60 (106.61)	6.75 (16.49)	0.001
Pre-operative eCCR ratio	1.13	1.11 (0.38)	1.27 (2.26)	1.25 (1.18)	1.23 (0.50)	0.001
Creatinine within 24 h before surgery (μmol/L)	102.69	100.31 (78.26)	98.75 (44.46)	104.65 (79.01)	107.90 (72.40)	0.138
Pre-operative creatinine	101.95	99.21 (72.06)	98.15 (40.11)	102.89 (68.31)	107.11 (70.80)	0.110
Creatinine 12–24 h after surgery	91.83	89.10 (74.79)	89.15 (45.57)	96.46 (68.71)	102.09 (65.22)	0.002
Creatinine 24 h after surgery	92.84	90.71 (73.06)	91.85 (45.48)	98.40 (70.21)	103.72 (65.53)	0.002
Creatinine at day 2 after surgery	102.72	99.13 (73.60)	96.19 (49.84)	104.08 (66.63)	107.76 (56.25)	0.006
Creatinine at day 4 after surgery	98.61	94.35 (75.25)	93.75 (57.22)	100.73 (74.97)	104.61 (69.26)	0.007
Maximum post-operative creatinine	111.03	108.53 (86.88)	110.96 (64.41)	119.15 (86.85)	126.22 (76.60)	< 0.001
Absolute difference in creatinine	9.08	9.32	12.81 (46.89)	16.26 (43.28)	19.11 (44.45)	< 0.001
Relative difference in creatinine	1.10	1.09	1.19	1.19 (0.79)	1.19 (0.40)	< 0.001
Percentual difference in creatinine	10.20	8.71	19.27	19.45 (79.05)	18.96 (39.91)	< 0.001
Urea within 24 h before surgery (mmol/L)	6.96	7.25	8.09	7.57 (3.27)	8.20 (3.76)	< 0.001
Pre-operative urea	6.98 (3.29)	7.34 (4.03)	8.25	7.51 (3.12)	8.52 (6.11)	< 0.001
Urea 12–24 h after surgery	7.24 (5.47)	8.11 (16.73)	8.55	8.19 (9.95)	9.37 (8.95)	< 0.001
Urea at day 2 after surgery	10.13 (23.15)	10.51 (25.40)	11.89	14.51 (35.13)	12.76 (24.00)	< 0.001
Urea at day 4 after surgery	8.49 (24.49)	9.32 (29.78)	10.74	14.14 (49.26)	11.35 (16.44)	< 0.001
Maximum CPB flow	4.63 (1.47)	4.12 (1.97)	3.96 (2.05)	4.02 (2.05)	4.14 (2.01)	< 0.001
Duration of perfusion	100.07 (38.64)	125.29 (48.12)	169.76 (73.36)	168.07 (50.43)	214.06 (77.13)	0.000
Aortic cross-clamp time	58.94 (25.42)	83.36 (32.28)	109.93 (53.19)	110.25 (31.48)	137.22 (52.95)	0.080
HR at start surgery	62.22 (12.90)	67.14 (14.00)	70.80 (17.52)	62.55 (13.90)	66.17 (16.06)	0.000
HR during perfusion	66.39 (57.83)	61.24 (55.15)	61.80 (53.20)	60.09 (57.94)	63.58 (61.49)	< 0.001
SBP at start surgery (mmHg)	113.54 (34.62)	108.81 (31.98)	102.25 (31.32)	109.36 (33.49)	105.28 (29.93)	0.002
SBP during perfusion	61.76 (21.39)	63.37 (22.60)	63.17 (20.39)	63.96 (20.10)	62.83 (22.03)	< 0.001
DBP at start surgery (mmHg)	64.81 (31.81)	61.87 (29.18)	60.45 (27.90)	60.51 (29.65)	58.87 (22.32)	0.012
DBP during perfusion	56.53 (18.09)	58.52 (18.88)	57.58 (17.00)	59.16 (17.66)	57.39 (17.37)	< 0.001
CVP at start surgery (mmHg)	12.58 (30.79)	11.96 (28.43)	13.88 (30.11)	12.89 (32.66)	12.32 (24.81)	< 0.001
CVP during perfusion	6.62 (8.31)	5.03 (9.45)	4.78 (15.07)	5.65 (5.60)	4.44 (7.75)	0.653
PaCO_2_ at start surgery (kPa)	5.02 (0.63)	5.08 (0.70)	5.03 (0.69)	5.07 (0.64)	5.01 (0.72)	< 0.001
PaCO_2_ during perfusion	5.04 (0.54)	5.17 (0.57)	5.18 (0.62)	5.09 (0.51)	5.13 (0.57)	0.010
PaCO_2_ at end surgery	4.84 (0.59)	4.87 (0.63)	4.99 (0.74)	4.89 (0.62)	5.04 (0.72)	< 0.001
PaO_2_ at start surgery (kPa)	21.49 (14.95)	22.11 (14.43)	22.03 (14.74)	20.65 (12.85)	19.81 (12.93)	< 0.001
PaO_2_ during perfusion	26.70 (10.88)	25.59 (10.36)	25.88 (9.82)	25.87 (9.37)	26.82 (10.52)	0.018
PaO_2_ at end surgery	17.79 (11.58)	22.27 (13.04)	21.93 (12.82)	21.30 (12.62)	20.37 (11.37)	0.001
SaO_2_ at start surgery (%)	0.98 (0.03)	0.98 (0.03)	0.98 (0.05)	0.98 (0.03)	0.98 (0.02)	< 0.001
SaO_2_ during perfusion	0.99 (0.03)	0.99 (0.05)	0.99 (0.05)	0.99 (0.03)	0.99 (0.06)	0.206
SaO_2_ end surgery	0.98 (0.03)	0.99 (0.04)	0.98 (0.04)	0.98 (0.04)	0.98 (0.02)	0.152
ICU stay (hours)	52.44 (163.21)	47.51 (138.81)	88.79 (216.86)	88.72 (260.41)	141.13 (267.53)	< 0.001
ESR within 24 h before surgery (mm/h)	20.61 (19.57)	18.63 (19.96)	20.21 (19.40)	22.23 (20.25)	23.01 (21.03)	< 0.001
Pre-operative ESR	20.85 (19.77)	17.98 (19.27)	19.22 (19.06)	21.22 (19.82)	23.15 (19.74)	< 0.001
LDH within 24 h before surgery (U/L)	227.71 (75.41)	248.34 (115.16)	259.90 (169.51)	235.06 (70.41)	228.79 (66.45)	< 0.001
Pre-operative LDH	228.65 (76.10)	250.27 (142.33)	273.05 (428.61)	236.90 (74.54)	230.82 (74.38)	< 0.001
LDH 12- 24 h after surgery	338.15 (273.89)	396.80 (179.67)	480.19 (484.17)	456.26 (497.74)	510.39 (662.83)	< 0.001
LDH at day 2 after surgery	338.30 (233.89)	388.29 (252.01)	461.39 (444.76)	446.04 (312.62)	474.68 (264.37)	< 0.001
LDH at day 4 after surgery	327.78 (882.49)	382.96 (703.42)	413.65 (329.29)	424.52 (461.76)	439.23 (340.88)	< 0.001
Maximum post-operative LDH	421.61 (896.25)	461.39 (377.02)	568.32 (731.73)	558.72 (709.25)	592.21 (543.21)	< 0.001
Blood glucose 0–6 h after surgery (mmol/L)	9.41 (2.46)	8.41 (2.48)	8.47 (2.84)	8.48 (2.73)	9.02 (2.70)	< 0.001
Blood glucose 6–12 h after surgery	10.22 (2.43)	9.56 (2.00)	9.49 (2.27)	9.67 (2.15)	9.57 (2.26)	< 0.001
Blood glucose 12–24 h after surgery	9.14 (2.48)	8.39 (2.07)	8.17 (2.21)	8.27 (2.13)	8.12 (2.08)	< 0.001
Maximum post-operative glucose	11.19 (4.37)	10.38 (3.84)	10.48 (2.58)	10.53 (2.24)	10.82 (2.69)	< 0.001
Hb within 24 h before surgery (mmol/L)	8.47 (1.09)	8.45 (1.06)	8.27 (1.20)	8.35 (1.00)	8.30 (1.11)	< 0.001
Pre-operative Hb	8.19 (1.36)	8.24 (1.78)	8.10 (1.65)	8.26 (2.44)	8.34 (2.90)	< 0.001
Hb 0–6 h after surgery	5.64 (0.73)	5.69 (0.76)	5.78 (0.82)	5.52 (0.76)	5.57 (0.84)	0.135
Hb 6–12 h after surgery	6.02 (0.85)	6.35 (1.16)	6.22 (0.93)	5.92 (0.84)	5.80 (0.89)	< 0.001
Hb 12–24 h after surgery	6.18 (0.78)	6.40 (0.85)	6.25 (0.88)	6.01 (0.77)	5.92 (0.80)	< 0.001
Hb at day 2 after surgery	6.31 (0.78)	6.26 (0.81)	6.09 (0.86)	6.01 (0.75)	5.92 (0.76)	< 0.001
Hb at day 4 after surgery	6.52 (0.87)	6.40 (1.33)	6.22 (0.89)	6.07 (0.81)	5.97 (0.82)	< 0.001
Minimum post-operative Hb	5.31 (0.69)	5.41 (0.73)	5.29 (0.77)	5.11 (0.65)	5.01 (0.70)	< 0.001
Leukocytes within 24 h before surgery (× 10^9^/L)	7.84 (2.73)	7.44 (2.70)	7.62 (3.32)	7.77 (3.22)	7.74 (2.18)	< 0.001
Pre-operative leukocytes	8.01 (2.96)	7.53 (2.63)	7.79 (3.12)	7.88 (2.99)	7.88 (2.46)	< 0.001
Leukocytes 12–24 h after surgery	13.95 (4.41)	13.71 (4.36)	13.79 (4.17)	13.57 (4.93)	13.49 (4.22)	< 0.001
Leukocytes at day 2 after surgery	17.08 (4.82)	15.79 (4.81)	15.99 (5.12)	16.14 (4.74)	16.49 (4.85)	0.051
Leukocytes at day 4 after surgery	11.52 (4.15)	10.00 (4.06)	10.93 (9.74)	10.99 (3.94)	11.96 (4.78)	< 0.001
Thrombocytes within 24 h before surgery (× 10^9^/L)	246.55 (73.47)	231.91 (67.44)	235.83 (72.42)	234.86 (69.37)	239.06 (72.70)	< 0.001
Pre-operative thrombocytes	238.69 (78.71)	224.72 (71.67)	230.34 (75.95)	230.41 (73.07)	233.71 (76.22)	< 0.001
Thrombocytes 0–6 h after surgery	152.85 (52.79)	131.80 (44.10)	132.02 (44.78)	129.11 (46.63)	131.97 (48.12)	< 0.001
Thrombocytes 6–12 h after surgery	171.17 (58.06)	149.14 (49.54)	141.39 (48.69)	136.37 (47.14)	140.20 (54.93)	< 0.001
Thrombocytes 12–24 h after surgery	174.48 (57.73)	151.22 (50.74)	141.85 (47.48)	136.92 (46.77)	138.73 (53.92)	< 0.001
ALAT within 24 h before surgery (U/L)	40.56 (35.46)	28.54 (26.10)	31.48 (29.11)	30.31 (28.08)	31.99 (26.70)	< 0.001
Pre-operative ALAT	40.80 (35.28)	28.86 (27.93)	33.58 (57.80)	30.40 (26.64)	33.25 (34.18)	< 0.001
ALAT 12–24 h after surgery	37.49 (79.01)	29.24 (37.05)	43.72 (160.67)	35.59 (135.12)	46.14 (198.66)	< 0.001
ALAT at day 2 after surgery	37.57 (146.05)	31.20 (88.67)	44.26 (123.93)	40.56 (168.31)	40.43 (105.37)	0.002
ASAT within 24 h before surgery (U/L)	32.72 (20.36)	29.50 (20.19)	31.83 (24.10)	28.95 (15.01)	31.08 (28.86)	0.169
Pre-operative ASAT	33.18 (24.15)	30.11 (24.14)	37.82 (166.12)	29.45 (15.34)	30.90 (18.47)	< 0.001
ASAT 12–24 h after surgery	59.82 (108.39)	71.96 (83.88)	112.66 (241.82)	98.08 (206.81)	121.96 (283.96)	< 0.001
ASAT at day 2 after surgery	53.36 (171.29)	58.70 (113.12)	92.51 (194.80)	89.26 (347.04)	90.90 (115.64)	0.011
ASAT at day 4 after surgery	55.12 (422.01)	54.44 (197.38)	68.11 (217.54)	72.37 (435.80)	71.26 (248.91)	< 0.001
Neutrophils 12–24 h after surgery (× 10^9^/L)	12.29 (3.86)	12.07 (3.86)	12.07 (3.80)	11.86 (3.96)	11.79 (3.82)	0.584
Monocytes 12–24 h after surgery (× 10^9^/L)	1.10 (1.73)	1.32 (2.07)	1.51 (2.25)	1.42 (2.33)	1.34 (2.20)	0.004
Lymphocytes 12–24 h after surgery (× 10^9^/L)	1.05 (2.05)	1.12 (1.86)	1.35 (2.60)	1.15 (1.93)	1.34 (3.11)	< 0.001
5-year mortality:						0.001
Alive	3890 (86.18%)	1382 (83.10%)	710 (80.32%)	642 (78.97%)	261 (71.12%)	< 0.001
Deceased	624 (13.82%)	281 (16.90%)	174 (19.68%)	171 (21.03%)	106 (28.88%)	
Minimum body temperature	31.71 (1.82)	31.20 (2.60)	30.76 (2.36)	31.23 (2.17)	30.89 (1.86)	
AKI staging						< 0.001
No AKI	3063 (67.86%)	1142 (68.67%)	584 (66.06%)	462 (56.83%)	199 (54.22%)	< 0.001^#^
Mild subclinical AKI	841 (18.63%)	268 (16.12%)	133 (15.05%)	145 (17.84%)	62 (16.89%)	
Moderate subclinical AKI	142 (3.15%)	51 (3.07%)	26 (2.94%)	37 (4.55%)	14 (3.81%)	
AKI 1–3	468 (10.37%)	202 (12.15%)	141 (15.95%)	169 (20.79%)	92 (25.07%)	
AKI 1	441 (9.77%)	191 (11.49%)	126 (14.25%)	157 (19.31%)	90 (24.52%)	
AKI 2	9 (0.20%)	6 (0.36%)	11 (1.24%)	6 (0.74%)	2 (0.54%)	
AKI 3	18 (0.40%)	5 (0.30%)	4 (0.45%)	6 (0.74%)	0 (0%)	

All values presented as mean (95% CI), and categorical variable with the percentage in parentheses.

*BMI* body mass index, *eCCR* estimated creatinine clearance, *CPB* cardio-pulmonary bypass, *HR* heart rate, *SBP* systolic blood pressure, *DBP* diastolic blood pressure, *CVP* central venous pressure, *PaCO*_*2*_ arterial CO_2_ pressure, *PaO*_*2*_ arterial oxygen pressure, *SaO*_*2*_ oxygen saturation, *ICU* intensive care unit, *ESR* erythrocyte sedimentation rate, *LDH* lactate dehydrogenase, *Hb* hemoglobin, *ALAT* alanine aminotransferase, *ASAT* aspartate aminotransferase, *AKI* acute kidney injury.

^#^Significance level presented is for AKI 1–3 combined, given that there are no patients in the mitral + coronary group with AKI 3.

### Machine learning analysis

**Figure 1:**
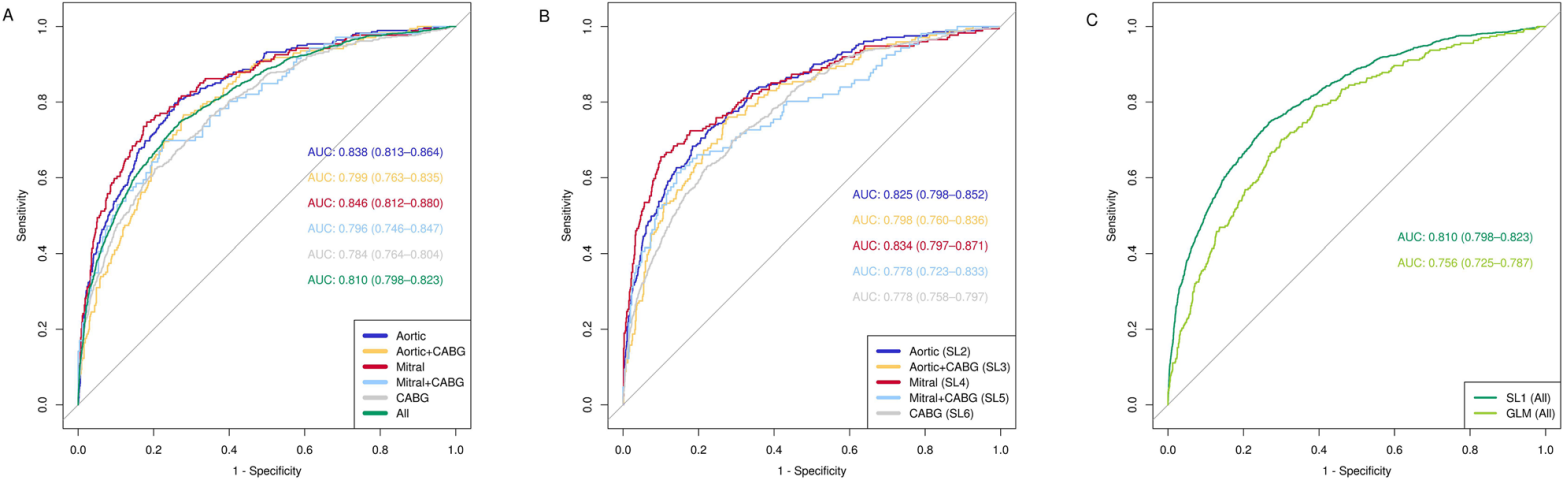
Plot of the receiver operating characteristic (ROC) curves and the respective areas under curve (AUCs) for the weighted Super Learner 1 for each of the 5 types of operation and for the whole cohort. Plot of the receiver operating characteristic (ROC) curves and the respective areas under curve (AUCs) for the weighted Super Learner and the generalized linear model (GLM) for the whole cohort. *SL* super learner, *CABG* coronary artery bypass grafting.

As a first step in the ML based prediction of long-term mortality, the ensemble was trained on the full cohort (SL1; Fig. 5, left part). ROC curves and their respective AUROCs were established for the full cohort and the different cardiac operation types (Fig. 1). SL1 achieved an AUROC of 0.810 [0.798–0.823]. When analyzed per operation type, the accuracy of SL1 was highest for solitary mitral valve (0.846) and solitary aortic valve operations (0.838), and lowest for solitary CABG (0.784) and mitral valve/CABG (0.796). In addition, the comparison between SL1 and the trained GLM showed that the SL1 significantly outperformed GLM (AUROC 0.756 [0.725–0.787]) for the full cohort (*P* = 0.0016; Fig. 1) as well as for solitary aortic valve and combined aortic valve and CABG (*P* < 0.01; Table 2 in the “Supplementary material”). Thus, SL1 produced sound long-term mortality prediction based on peri-operative routinely collected patient and operation data.

Next, we performed a similar analysis based on SL training per operation type, by making five training sets using 80% of the relevant patients to train five weighted ensembles (SL2–SL6). Comparison of AUROCs between SL1 versus SL2–6, showed identical ranking for specific operation subgroups. Predictive performance between the models generated by SL1 compared those from SL2 to SL6 did not differ (Fig. 1; Table 2 in the “Supplementary material”). SL3 and SL4 also outperformed GLM (*P* < 0.01; Table 4 in the “Supplementary material”). Lastly, because of its potential ability to identify patients at high risk prior to surgery, we examined the predictive performance when only pre-operative data are included. As expected, the model trained only on pre-operative data showed inferior performance to the full peri-operative model (AUROC 0.718 [0.687–0.749], *P* < 0.01, Fig. 12 in the “Supplementary material”).

### Calibration, sensitivity analysis and adjusted risk thresholds based on predicted probability of mortality

**Figure 2:**
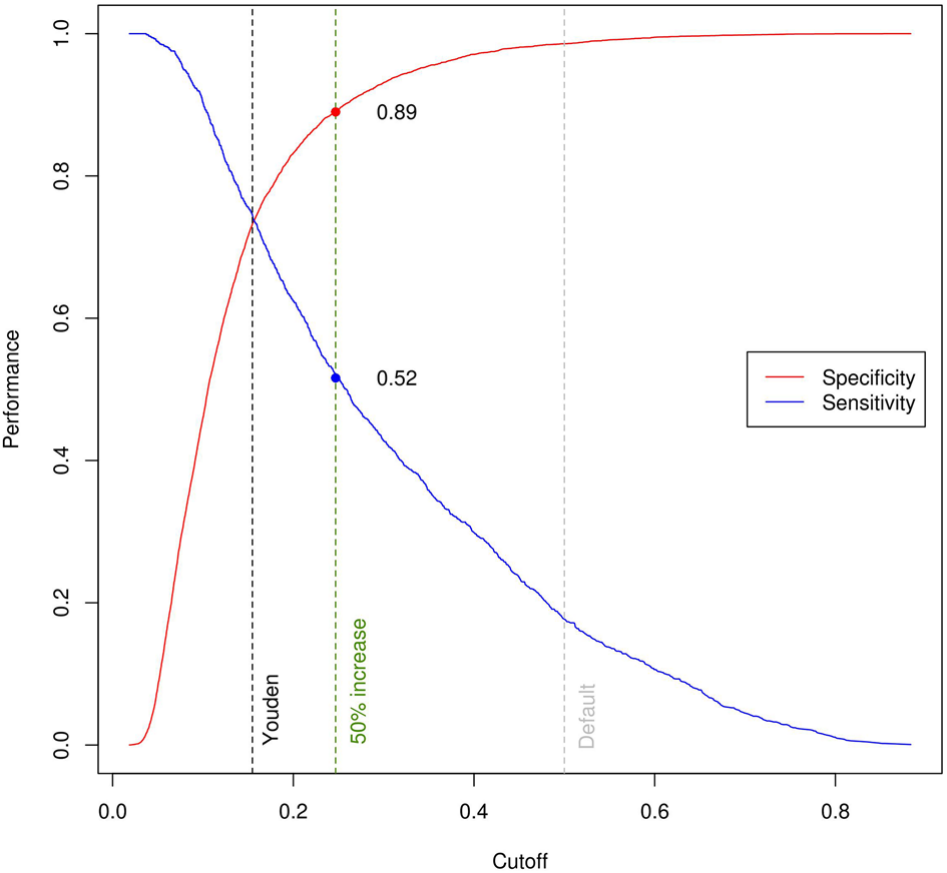
Specificity (blue) and sensitivity (red) values across all possible thresholds for all operations combined. The default 0.50 threshold is marked in grey, the threshold based on the maximized Youden index in black, and the threshold representing a 50% increase in mortality risk in green.

**Figure 3:**
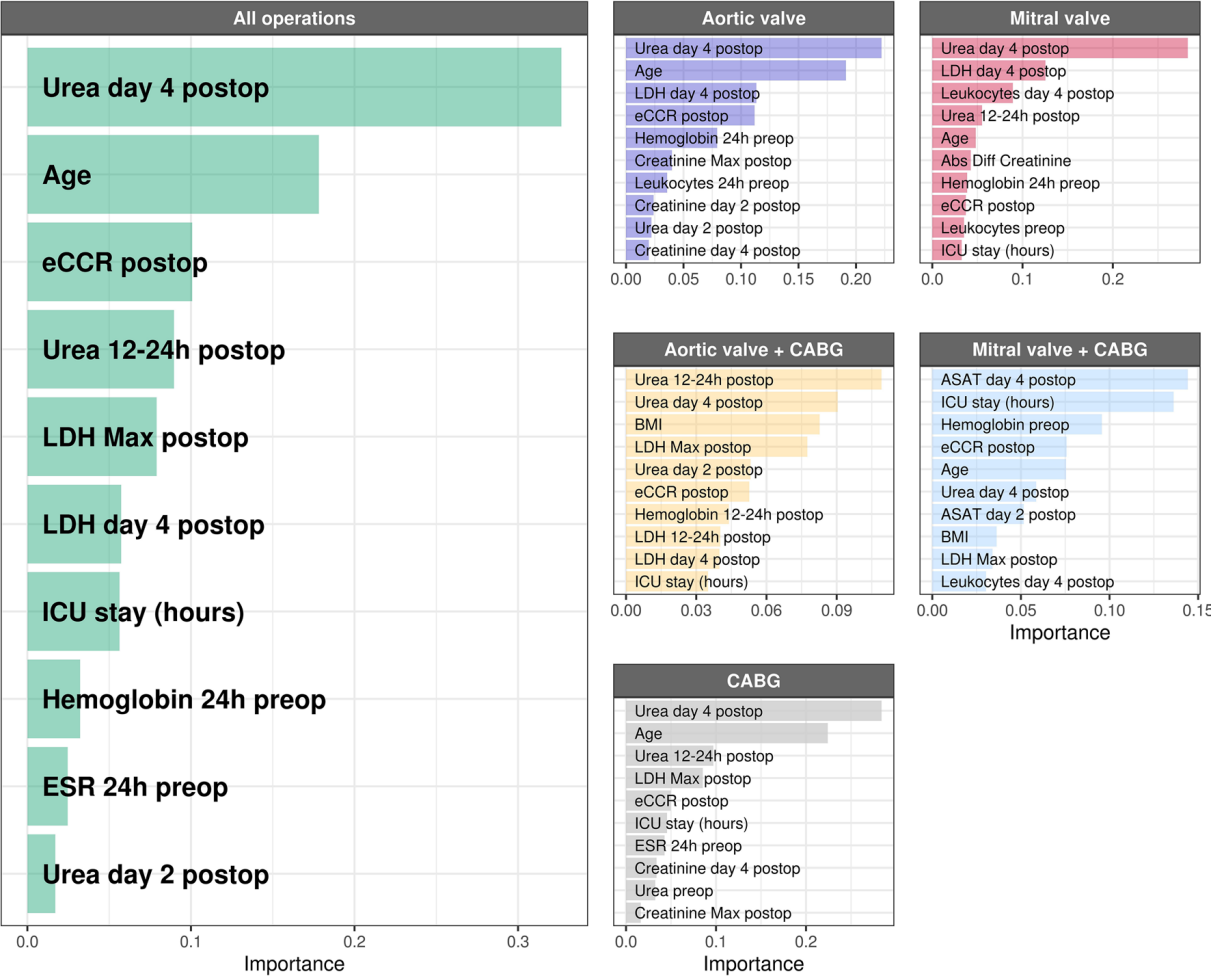
Top ten predictor variables for all types of operations combined. Variable coefficients indicate how much each parameter contributes to the outcome. *eCCR* estimated creatinine clearance, *LDH* lactate dehydrogenase, *ESR* erythrocyte sedimentation rate, *ICU* intensive care unit, *ASAT* aspartate transaminase, *BMI* body mass index.

**Figure 4:**
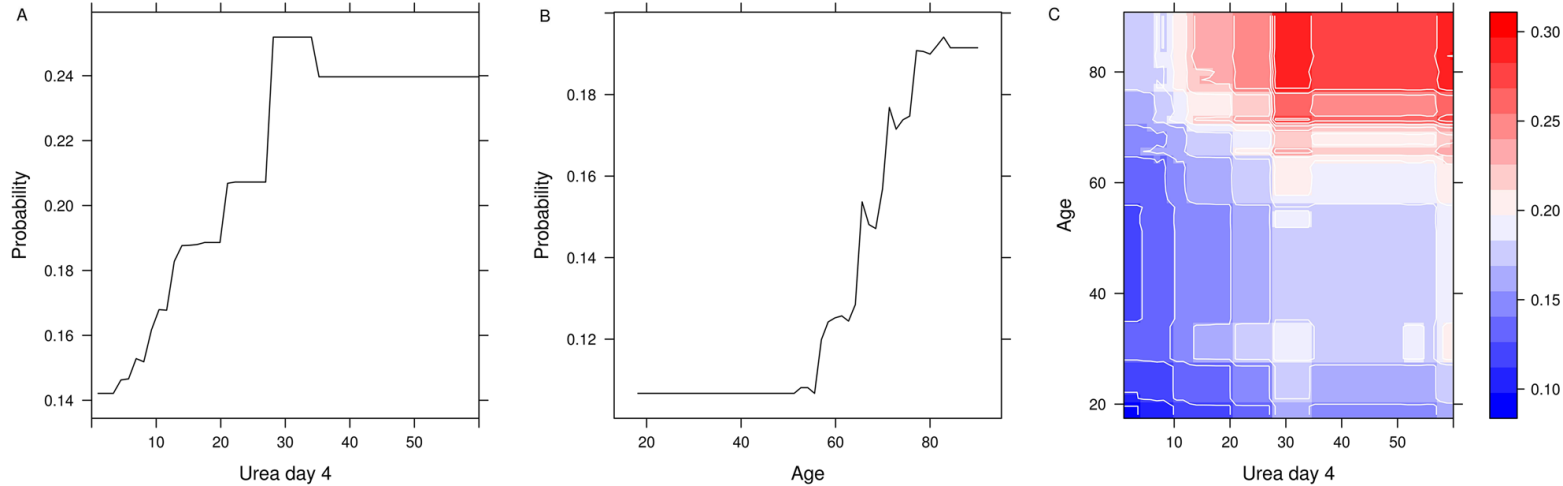
Partial dependence plots of urea at postoperative day 4 and age. Partial dependence plots of urea at postoperative day 4 against age. The vertical bar represents predicted risk (blue to red, low to high).

**Figure 5:**
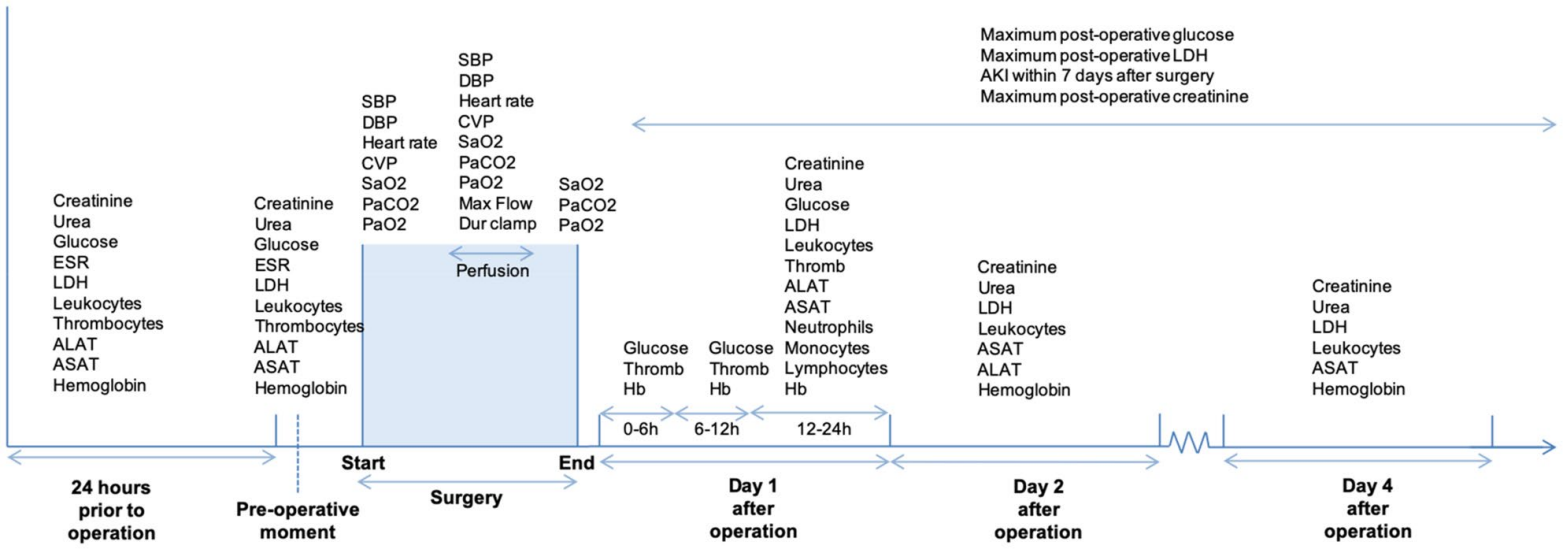
Timeline of clinical measurements before, during, and after cardiac operation, in the intensive care unit (day 1 after operation), day 1 in the ward (day 2 after operation), and day 3 in the ward (day 4 after operation). Patient characteristics are not included here, but described in detail in Table 1. *Dur CA* duration of cardiac arrest, *Dur clamp* duration of aortic cross-clamp, *Hb* hemoglobin, *ASAT* aspartate aminotransferase, *ALAT* alanine aminotransferase, *Thromb* thrombocytes, *ESR* erythrocyte sedimentation rate, *LDH* lactate dehydrogenase, *CVP* central venous pressure, *PaCO*_*2*_ arterial carbon dioxide partial pressure, *SaO*_*2*_ oxygen saturation, *PaO*_*2*_ arterial oxygen partial pressure, *SBP* systolic blood pressure, *DBP* diastolic blood pressure.

**Figure 6:**
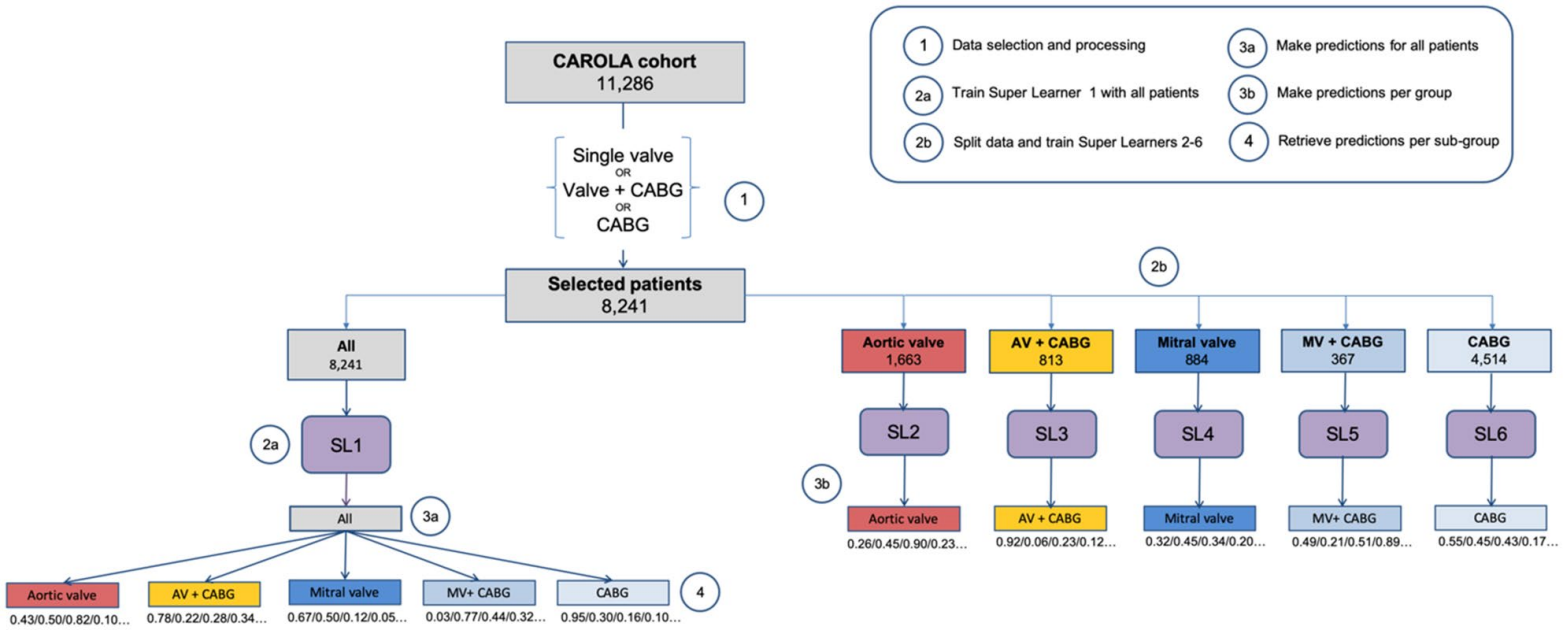
Diagram of the steps involved in data analysis: data split, algorithm training, and outcome prediction using different Super Learner ensembles. On the left, the process of training the single Super Learner on data of the whole cohort (n = 8241), obtaining the pooled predicted probabilities, and retrieving the group-specific probabilities to calculate the performance measures for each type of operation. On the right, the process of splitting the data into five groups, one per operation type, and training a different super learner on data from one type of operation only. *SL* super learner, *AV* aortic valve, *MV* mitral valve, *CABG* coronary artery bypass grafting.

Calibration of SL1 and SL2–6 was good for most models (Table 5 and Figs. 1–11 of the “Supplementary material”). Using the adjusted thresholds based on the Youden index and on a 50% increased risk of mortality lead to improved model sensitivity and specificity (Fig. 2). For all operations, the thresholds based on the Youden index approximated the baseline absolute mortality risk. Compared to the default threshold of 50% mortality risk, both the thresholds based on the Youden index and the thresholds defined by a 50% increased risk of mortality increased sensitivity substantially for all types of operation (Tables 6–15 of the “Supplementary material”). For the Youden index thresholds, this was paired with a steeper decrease in specificity than for the thresholds at 50% increased risk of mortality. As Table 2 shows, the threshold representing 50% increase in risk improved the number of patients correctly classified as “non-survivor” for all types of operation. The largest increase in correctly classified “non-survivors” was observed for aortic valve, CABG, combined aortic valve and CABG, and for all operations combined (3-, 4.7-, 2.2-, and 3-fold increase).

**Table 2:** Percentage of correctly classified cases in survivors and non-survivors per operation type for SL1 predictions using the default and 50% increase in risk thresholds.

	Predictions matching actual patient outcome (%)	
	Survivors (%)	Non-survivors (%)
**Aortic valve**		
With default threshold	98.8	18.1
With 50% increased risk threshold	90.5	53.0
Difference	**− 8.3**	**+ 34.9**
**Mitral valve**		
With default threshold	96.8	34.5
With 50% increased risk threshold	89.7	59.8
Difference	**− 7.1**	**+ 25.3**
**CABG**		
With default threshold	99.2	10.4
With 50% increased risk threshold	88.9	47.9
Difference	**− 9.3**	**+ 37.5**
**Aortic + CABG**		
With default threshold	97.0	19.9
With 50% increased risk threshold	88.8	43.3
Difference	**− 8.2**	**+ 23.4**
**Mitral + CABG**		
With default threshold	96.9	28.3
With 50% increased risk threshold	95.4	34.9
Difference	**− 1.5**	**+ 6.6**
**All operations combined**		
With default threshold	98.6	17.7
With 50% increased risk threshold	89.4	51.6
Difference	**− 9.2**	**+ 33.9**

### Variable importance analysis

Unexpectedly, variable importance analysis of all operations combined (n = 8142) revealed serum urea at day 4 after operation as the top predictor variable for 5-year mortality (Fig. 3). Serum urea was also found the top predictor in all operation types, except for the smallest group (n = 367), combined mitral valve and CABG operations. Other important predictive variables included patient age, serum urea at other time points, indicators of kidney function, and serum markers for organ damage and inflammation. To better illustrate the impact of the changes in these variable and possible interactions, we constructed probability plots of the two highest ranking variables in all patients (Fig. 4). Mortality risk steeply increased from day 4 urea levels of 10 mmol/L, reaching a plateau at 30 mmol/L denoting a 50% increase in absolute risk compared to baseline. Likewise, mortality risk gradually increased between 60 and 80 years of age. Figure 4 illustrates the combined effect of serum urea day 4 and age on mortality risk.

## Discussion

This study shows that ensemble ML analysis achieves a high accuracy in predicting 5-year mortality in a cohort of 8241 patients with CABG and/or valve operations. Moreover, variable importance analysis revealed early postoperative urea as a novel and strong predictor of mortality in all types of cardiac operations. Furthermore, methodologically, a more targeted approach of training the algorithms on sub-groups instead of the full cohort did not significantly improve mortality prediction.

We demonstrated that using an ensemble algorithm with a combination of pre-operative, intra-operative, and first week post-operative data, achieves high accuracy in predicting 5-year mortality after different types of cardiac operations. These findings extend a previous study where we demonstrated the superiority of individual ML models compared to classical multivariable analysis in identifying patients at increased risk of long-term mortality after CABG^7^. Here, we reaffirm these findings using ensemble ML and data from different types of cardiac operations. Using peri-operative data, we achieved similar accuracy to a recently developed ML-based risk algorithm for prediction of 1- to 24-month mortality following major surgery^15^. Compared to other models that predict mortality specifically after cardiac surgery, the ensemble achieved superior performance^8^.

The application of algorithms such as the one we developed to pre-operative data would possibly predict patients at the highest risk of long-term complications prior to surgery. Expectedly, analysis of pre-operative data in the XGBoost model decreased performance significantly, which could be partly due to the limited set of pre-operative data available in our cohort, or to the lower frequency of the outcome (long-term mortality as opposed to short-term post-operative complications). Yet, it should be noted that the model’s performance using our restricted set of pre-operative data has comparable predictive power as currently used clinical scores^8^.

Methodologically, our study contributed to the discussion on the need of conducting predictive studies on operation-specific cohorts. Results from previous studies suggest that algorithms trained on pooled data from patients undergoing different types of surgeries were accurate in predicting outcomes for all these types of operations. In keeping, our findings show that both the model trained with the full cohort, and the models trained with the individual cardiac operation subgroups showed a good performance in predicting long-term mortality after aortic and mitral valve operations. This finding further questions the need to conduct ML analyses on operation-specific cohorts. Specifically, including full cohorts may lead to better model performance analyses due to the greater amount of data.

Additionally, by providing risk predictions at individual level, ML algorithms allow for the adjustment of the sensitivity and specificity of each model for different clinical settings^15^. Balancing sensitivity and specificity in the context of mortality risk predictions can be challenging. Lowering the prediction threshold may lead to excessive over-diagnosing and increase in healthcare costs. However, especially in populations with relatively low mortality rates such as cardiac surgery patients, a too high threshold would miss too many “non-survivors”. Here, we demonstrated that using a 50% increase in absolute risk of mortality as cut-off provides a favorable trade-off between false positives and true negatives, as previously shown in similar large studies predicting postoperative mortality and mortality in intensive care patients^15,16^. Validation of this approach merits further investigation, and may facilitate the translation of an algorithm’s good predictive performance into a clinically useful patient risk stratification tool^17^.

Variable importance analysis identified postoperative urea as the strongest predictor of 5-year mortality. This is consistent with our previous findings in a CABG-only population^7^. Yet, literature on the possible role of urea as a mortality predictor in cardiac operations is scarce^7^. Preoperative urea values above 10 mmol/L have been found to be associated with increased 30-day mortality risk after CABG and with increased risk of stroke in the 10 days after cardiac operations^18,19^. It should also be noted that, in heart failure patients, increased urea levels have been associated with derangements in cardiac output and renal perfusion^20,21^. These are, in turn, strongly related to patients’ overall performance status and prognosis, with both urea and the urea/creatinine ratio being known prognostic predictors^22^. In the context of this study, increased urea may originate from excess production and/or impaired excretion, yet mechanistic insight remains elusive. Possibly, urea production may be increased by mitochondrial dysfunction, caused by ischemia/reperfusion and increased systemic inflammatory response after cardiopulmonary bypass and surgical trauma^23^. Mitochondrial dysfunction may be amplified through excess reactive oxygen species (ROS) following accumulation of succinate during ischemia^24,25^. Additionally, recent evidence indicates that high urea levels generate ROS^26^. Furthermore, renal excretion of urea may decrease in response to kidney injury. Thus, urea likely reflects the compound pathological state of different organ systems, rather than just kidney function.

Lastly, this study also has some limitations to consider. Being a single center study, our findings need confirmation by external validation. Further, our analysis is limited to the variables in the CAROLA database. Detailed co-morbidity information, for instance, could help further improve model performance, especially for the CABG sub-group. Additionally, variable importance analysis as such does not provide directionality and assumptions about effect size between the variables and the outcome cannot be made directly. Finally, the current ensemble ML is not suited to use high-frequency, high-volume data, such as continuous intraoperative measurements of blood pressure, heart rate, oxygen saturation or temperature. Therefore, a study including algorithms suitable for such analysis, such as recurrent neural networks, is a logical follow-up.

In conclusion, ML analysis of 88 routinely collected peri-operative data achieved a high accuracy in predicting 5-year mortality after different cardiac operations in this large study of 8241 patients. A targeted approach of training the algorithms on sub-groups instead of the full cohort did not improve model performance. Moreover, variable importance analysis showed early postoperative urea as a novel and strong predictor of mortality in all types of cardiac operations. Similar studies enabling the identification of modifiable risk factors and providing individual patient predictions may form a first step towards facilitating personalized clinical interventions to improve patient care.

## Methods

The electronic Cardiothoracic Anesthesiology Registry (CAROLA) comprises extensive prospective data of all adult patients who underwent first-time valve operation, CABG, or a combination of both between 1997 and 2017 in the University Medical Centre Groningen (UMCG), the Netherlands. The total number of patients is 11,286. This database study was approved by the Medical Ethical Committee of the UMCG, and the requirement to obtain informed consent was waived (waiver: METC#2010/118). All analyses were performed in accordance with relevant guidelines and regulations.

### Patient population and outcome

Only patients who underwent valve operation, either solitary or combined with coronary artery bypass grafting (CABG), or solitary CABG, with cardiopulmonary bypass (CPB) were included (n = 8241). There were 1663 patients in the combined aortic and coronary group, 367 in the combined mitral and coronary group, 884 in the solitary mitral group, 813 in the solitary aortic group, and 4514 in the CABG-only group. Mortality data were obtained in November 2017 from the Dutch Municipal Personal Records Database comprising actual and reliable data of all citizens within the Netherlands.

### Data selection and pre-processing

The dataset includes patient characteristics, peri-operative hemodynamic, CPB, respiratory and organ function data and blood values collected at different time points indicated in Fig. 5. Because for some patients referred from other hospitals the stay in our center was limited to the immediate peri-operative phase, a variable pattern of missing data was observed. Multivariate imputation by chained equations was performed on the set of variables with at least 50% non-missing data^27^. The final dataset without missing data consisted of 88 predictor variables and 5-year mortality as the outcome variable (Table 1). Baseline serum creatinine measurements was defined as the closest to the start of operation. Patients were classified for post-operative AKI 0–3 within the 7 days after operation according to the AKIN classification^3^*.*

### Statistical analysis

#### The Super Learner, selected candidate algorithms, and hyper-parameter tuning

The Super Learner algorithm is a generalization of the stacking algorithms developed by Breiman^28^, which combines a set of candidate algorithms to make k-fold-cross-validated predictions^9,29^. In this process, the dataset is divided into k mutually exclusive and exhaustive subsets, with one set serving as a validation set, while the others are used for training each candidate algorithm^14^. This means that each patient is used only once in the validation set, and included in the training set for all other rounds. For each candidate learner, k risks are calculated and averaged into a “cross-validated risk”. Subsequently, the learners with the minimal risk are selected, applied to the entire dataset and included in the new weighted estimator (the SL), that attributes a relative coefficient to each of the learners. Those which reduce the calculated risk the most, will contribute to the final weighted prediction. Moreover, the SL presents individual patient predicted probabilities for 5-year mortality per ensemble. Five candidate algorithms were included in the SL: support Bayesian additive regression trees (BART), extremely randomized trees, elastic net, support vector machine, and extreme gradient boosted machine (XGBoost). Details of these five algorithms can be found in the “Supplementary material”. Since the performance of an algorithm varies greatly depending on its hyper-parameters and can be substantially improved by tuning, multiple hyper-parameter combinations were generated for each candidate algorithm. Details of each of these algorithms including the hyper-parameters, the tuning process, and final values are described in the “Supplementary material”. A 10-fold cross-validated generalized linear regression model (GLM) was trained on data from the full cohort for use as baseline comparison of the SL’s performance. Lastly, to test the performance of a model using only pre-operative data in predicting post-operative outcomes, a 10-fold cross-validated XGBoost model was trained on data from the full cohort.

#### Model training

Two distinct training procedures for the SL were carried out (Fig. 6). First, one of the ensembles (SL1) was trained using the full cohort of 8241 patients. Secondly, the cohort was split into five different groups according to operation type, with one ensemble trained on data from each group (SL2–SL6). All six ensembles included the same candidate algorithms, and the same hyper-parameter configurations. Performance of two different approaches were assessed by comparison of the 10-fold cross-validated area under the receiver operated characteristic curve (AUROC), with a 95% confidence interval, for each of the weighted SL’s. Differences in the performance between SL’s and between SL1 and the GLM were assessed with DeLong’s nonparametric test for the difference in areas under the curve^30^.

#### Calibration, sensitivity analysis and adjusted risk thresholds based on predicted probability of mortality

Calibration plots and calibration indices (ECI)^31^ for all models are provided in the “Supplementary material”. Model performance metrics described above were obtained in a 2-step procedure: first using a default threshold to maximize the AUROC, and then using adjusted thresholds to optimize sensitivity and specificity. This process of tuning the operating points of the ROC using different risk thresholds depending on the requirements of a specific clinical setting has been previously shown to optimize model sensitivity and specificity for mortality prediction^15^. In the first step, a default threshold of 0.50 was used, where patients are classified as “non-survivors” if the predicted probability of mortality is greater than 50%. This is the standard threshold used to maximize algorithm performance during training. After this, a second and third risk thresholds were defined. The second one was calculated based on the maximized Youden index, which provides a balance between sensitivity and specificity^15^. The third one was based on the actual long-term mortality rate of each of the surgical sub-groups, and corresponds to a 50% increase in the absolute risk of mortality. We opted for this value as it represents a clinically relevant increase that could justify intervention. The confusion matrix, sensitivity, and specificity for each of the thresholds are reported in the “Supplementary material”.

#### Variable importance analysis

Variable importance measures aim at estimating the contribution of predictor variables to changes in the outcome^32^. The greater the association between each feature and the outcome, the greater the decrease in accuracy upon its removal, and the higher its reported importance^32^. We determined the variable importance of all routinely measured peri-operative clinical parameters in our cohort by training the best performing individual algorithm included in the ensemble—the XGBoost model—using the same hyper-parameter configurations as in the SL. The coefficients for the top ten features for each operation type, as well as for all operations combined, are presented.

All analyses were performed using R version 3.6.2 (The R Foundation for Statistical Computing; Vienna, Austria) for Ubuntu 16.04 LTS. Data are expressed as mean (95% confidence interval), and categorical as percentages. A *P* value < 0.05 was accepted as a statistically significant difference.

## Supplementary Information

Supplementary Information.
