# Supplementary material for: Ensemble machine learning prediction and variable importance analysis of 5-year mortality after cardiac valve and CABG operations
Source: Sci Rep. 2021 Feb 10;11:3467. doi: 10.1038/s41598-021-82403-0 (PMC7876023; doi:10.1038/s41598-021-82403-0)
Supplement: Supplementary file 1 — Supplementary Information. [file 41598_2021_82403_MOESM1_ESM.pdf]

Supplementary material

Ensemble machine learning prediction and variable importance analysis of 5-year mortality after cardiac valve and CABG operations

José Castela Forte BSc (Hon)* [1,2,7] (ORCID ID: 0000-0001-9273-0702), Hubert E. Mungroop M.D.[2], Fred de Geus Ph.D.[2], Maureen L. van der Grinten[7], Hjalmar R. Bouma M.D., Ph.D.[1,3], Ville Pettilä M.D., Ph.D.[4], Thomas W.L. Scheeren M.D.,Ph.D.[2], Maarten W.N. Nijsten M.D.,Ph.D.[5], Massimo A. Mariani M.D.,Ph.D.[5], Iwan C.C. van der Horst M.D.,Ph.D.[7,8], Robert H. Henning M.D.,Ph.D.[1], Marco A. Wiering Ph.D.[6], Anne H. Epema M.D.,Ph.D.[2]

Content of supplementary material

- Data descriptives tables (table 1).
- Tables of P-values of the DeLong test for difference in areas under the curve for prediction performance of the different Super Learners (tables 2-4).
- Calibration plots and ECI coefficients for all models (table 5 and figures 1-11).
- Super Learner confusion matrices (tables 6-13).
- Specificity and sensitivity per operation with adjusted risk thresholds (tables 14-15).
- Peri-operative and pre-operative model comparison (figure 12)
- Detailed explanation of the Super Learner, and model hyper-parameter definitions and tuning.

Table 1. Descriptives table per outcome (Survivors vs Non-survivors)

	Survivors	Non-survivors	p-value
	N=6885	N=1356	
**BMI**	27.1 (27.0-27.2)	27.1 (26.9-27.4)	0.911
**Gender:**			0.006
**Female**	2077 (30.17%)	461 (34.00%)	
**Male**	4808 (69.83%)	895 (66.00%)	
**Age**	65.36 (65.09-65.63)	71.08 (70.60-71.56)	<0.001
**Pre-operative eCCR**	73.63 (72.33-74.92)	63.90 (60.52-67.28)	<0.001
**Post-operative eCCR**	69.55 (68.92-70.18)	53.99 (52.66-55.33)	<0.001
**Per-operative eCCR decrease**	4.077 (2.779-5.375)	9.909 (6.619-13.20)	0.001
**Pre-operative eCCR ratio**	1.115 (1.092-1.138)	1.376 (1.263-1.489)	<0.001
**Creatinine within 24 hours before surgery (μmol/L)**	97.53 (55.82)	125.98 (116.21)	<0.001
**Pre-operative creatinine**	97.29 (95.95-98.62)	121.75 (116.6-126.9)	<0.001
**Creatinine 12-24 hours after surgery**	86.84 (85.53-88.15)	117.64 (112.30-123.0)	<0.001
**Creatinine 24 hours after surgery**	88.18 (86.89-89.48)	119.50 (114.00-125.0)	<0.001
**Creatinine at day 2 after surgery**	96.40 (95.09-97.71)	128.35 (123.2-133.5)	<0.001
**Creatinine at day 4 after surgery**	91.85 (90.59-93.12)	127.42 (121.8-133.0)	<0.001
**Maximum post-operative creatinine**	104.1 (102.6-105.6)	151.91 (145.3-158.6)	<0.001
**Absolute difference in creatinine**	6.85 (6.14-7.56)	30.16 (26.20-34.10)	<0.001
**Relative difference in creatinine**	1.09 (1.07-1.10)	1.29 (1.22-1.36)	<0.001
**Percentual difference in creatinine**	8.9 (7.4-10.3)	28.9 (22.3-35.5)	<0.001
**Urea within 24 hours before surgery (mmol/L)**	6.93 (6.86-6.99)	8.94 (8.69-9.19)	<0.001
**Pre-operative urea**	6.99 (6.91-7.08)	8.90 (8.64-9.16)	<0.001
**Urea 12-24 hours after surgery**	7.32 (7.09-7.55)	9.92 (9.38-10.46)	<0.001
**Urea at day 2 after surgery**	10.48 (9.88-11.08)	13.27 (12.01-14.54)	<0.001
**Urea at day 4 after surgery**	8.72 (8.06-9.37)	14.00 (11.99-16.00)	<0.001
**Maximum CPB flow**	4.34 (4.30-4.38)	4.54 (4.45-4.62)	<0.001
**Duration of perfusion**	120.7 (119.4-122.0)	143.4 (139.5-147.3)	<0.001
**Aortic cross-clamp time**	76.26 (75.33-77.19)	86.14 (83.67-88.61)	<0.001
**HR at start surgery**	64 (64-64)	66 (65-67)	<0.001
**HR during perfusion**	64 (63-66)	64 (61-67)	0.880
**SBP at start surgery (mmHg)**	111 (110-111)	111 (109-113)	0.865
**SBP during perfusion**	62 (62-63)	63 (62-64)	0.374
**DBP at start surgery (mmHg)**	63 (63-64)	61 (60-63)	0.012

**DBP during perfusion**	57 (57-58)	57 (56-58)	0.857
**CVP at start surgery (mmHg)**	12 (11-13)	15 (13-17)	0.005
**CVP during perfusion**	6 (6-6)	6 (5-7)	0.661
**PaCO2 at start surgery (kPa)**	5.02 (5.00-5.04)	5.13 (5.09-5.17)	<0.001
**PaCO2 during perfusion**	5.19 (5.08-5.10)	5.09 (5.06-5.12)	0.878
**PaCO2 at end surgery**	4.86 (4.85-4.87)	4.95 (4.91-4.98)	<0.001
**PaO2 at start surgery (kPa)**	21.6 (21.2-21.9)	21.3 (20.5-22.1)	0.553
**PaO2 during perfusion**	26.1 (25.9-26.3)	27.4 (26.8-28.0)	<0.001
**PaO2 at end surgery**	19.4 (19.1-19.7)	20.7 (20.0-21.4)	0.001
**SaO2 at start surgery (%)**	0.98 (0.98-0.98)	0.98 (0.98-0.99)	0.015
**SaO2 during perfusion**	0.99 (0.99-0.99)	0.99 (0.99-0.99)	0.353
**SaO2 at end surgery**	0.98 (0.98-0.98)	0.98 (0.98-0.98)	0.148
**ICU stay (hours)**	47.49 (44.28-50.70)	141.0 (123.7-158.2)	<0.001
**ESR within 24 hours before surgery (mm/hour)**	18.93 (18.49-19.36)	28.10 (26.82-29.39)	<0.001
**Pre-operative ESR**	18.78 (18.34-19.21)	27.64 (26.37-28.91)	<0.001
**LDH within 24 hours before surgery (U/L)**	233.0 (230.9-235.1)	252.0 (244.7-259.2)	<0.001
**Pre-operative LDH**	233.9 (231.7-236.0)	263.2 (244.1-282.3)	0.003
**LDH 12- 24 hours after surgery**	365.3 (359.2-371.4)	482.1 (449.3-514.9)	<0.001
**LDH at day 2 after surgery**	356.9 (352.1-361.8)	486.7 (459.7-513.7)	<0.001
**LDH at day 4 after surgery**	323.2 (314.7-331.8)	562.7 (474.8-650.6)	<0.001
**Maximum post-operative LDH**	413.6 (404.2-422.9)	735.3 (647.5-823.2)	<0.001
**Blood glucose 0-6 hours after surgery (mmol/L)**	8.9 (8.8-9.0)	9.4 (9.2-9.5)	<0.001
**Blood glucose 6-12 hours after surgery**	9.9 (9.8-9.9)	10.1 (10.0-10.3)	0.003
**Blood glucose 12-24 hours after surgery**	8.7 (8.7-8.8)	8.9 (8.8-9.0)	0.032
**Maximum post-operative glucose**	10.8 (10.7-10.9)	11.4 (11.2-11.5)	<0.001
**Hb within 24 hours before surgery (mmol/L)**	8.507 (8.483-8.532)	8.000 (7.931-8.066)	<0.001
**Pre-operative Hb**	8.285 (8.245-8.325)	7.799 (7.707-7.890)	<0.001
**Hb 0-6 hours after surgery**	5.660 (5.642-5.677)	5.581 (5.540-5.623)	0.001
**Hb 6-12 hours after surgery**	6.124 (6.101-6.146)	5.918 (5.874-5.963)	<0.001
**Hb 12-24 hours after surgery**	6.239 (6.220-6.259)	6.038 (5.996-6.080)	<0.001
**Hb at day 2 after surgery**	6.258 (6.239-6.277)	6.093 (6.051-6.136)	<0.001
**Hb at day 4 after surgery**	6.440 (6.416-6.464)	6.163 (6.118-6.209)	<0.001
**Minimum post-operative Hb**	5.325 (5.308-5.341)	5.146 (5.110-5.183)	<0.001
**Leukocytes within 24 hours before surgery ($x10^9$/L)**	7.6 (7.6-7.7)	8.3 (8.1-8.4)	<0.001
**Pre-operative leukocytes**	7.8 (7.7-7.8)	8.4 (8.2-8.6)	<0.001
**Leukocytes 12-24 hours after surgery**	13.8 (13.7-13.9)	13.8 (13.6-14.1)	0.884

**Leukocytes at day 2 after surgery**	16.6 (16.4-16.7)	16.7 (16.4-16.9)	0.491
**Leukocytes at day 4 after surgery**	10.8 (10.7-10.9)	12.6 (12.2-13.0)	<0.001
**Thrombocytes within 24 hours before surgery (x10^9/L)**	240 (238-241)	247 (242-251)	0.003
**Pre-operative thrombocytes**	233 (231-235)	239 (234-244)	0.017
**Thrombocytes 0-6 hours after surgery**	143 (142-144)	145 (141-148)	0.286
**Thrombocytes 6-12 hours after surgery**	160 (159-161)	151.5 (148-155)	<0.001
**Thrombocytes 12-24 hours after surgery**	163 (161-164)	152 (149-156)	<0.001
**ALAT within 24 hours before surgery (U/L)**	36.42 (35.64-37.21)	32.43 (30.95-33.92)	<0.001
**Pre-operative ALAT**	36.77 (35.97-37.57)	33.61 (31.03-36.19)	0.022
**ALAT 12-24 hours after surgery**	34.02 (32.63-35.41)	50.25 (39.19-61.32)	0.004
**ALAT at day 2 after surgery**	33.20 (31.23-35.18)	58.88 (44.31-73.44)	0.001
**ASAT within 24 hours before surgery (U/L)**	31.55 (31.08-32.03)	31.39 (30.08-32.71)	0.821
**Pre-operative ASAT**	32.01 (31.49-32.53)	35.53 (28.30-42.76)	0.341
**ASAT 12-24 hours after surgery**	66.50 (64.62-68.38)	114.98 (97.99-132.0)	<0.001
**ASAT at day 2 after surgery**	56.11 (52.77-59.46)	103.15 (85.34-121.0)	<0.001
**ASAT at day 4 after surgery**	43.99 (40.04-47.94)	133.98 (90.91-177.0)	<0.001
**Neutrophils 12-24 hours after surgery (x10^9/L)**	12.17 (12.08-12.26)	12.12 (11.91-12.34)	0.717
**Monocytes 12-24 hours after surgery (x10^9/L)**	1.213 (1.168-1.258)	1.330 (1.213-1.447)	0.067
**Lymphocytes 12-24 hours after surgery (x10^9/L)**	1.076 (1.030-1.121)	1.355 (1.197-1.513)	0.001
**Minimum Body Temperature**	31.48 (31.44-31.53)	31.10 (30.97-31.23)	<0.001
**Type operation:**			<0.001
**CABG**	3890 (56.50%)	624 (46.02%)	
**Aortic valve**	1382 (20.07%)	281 (20.72%)	
**Mitral valve**	710 (10.31%)	174 (12.83%)	
**Aortic + coronary**	642 (9.32%)	171 (12.61%)	
**Mitral + coronary**	261 (3.79%)	106 (7.82%)	
**AKI staging:**			<0.001
**No AKI**	4760 (69.14%)	690 (50.88%)	
**Mild subclinical AKI**	1222 (17.75%)	227 (16.74%)	
**Moderate subclinical AKI**	236 (3.43%)	34 (2.51%)	
**AKI 1**	639 (9.28%)	366 (26.99%)	
**AKI 2**	14 (0.20%)	20 (1.47%)	
**AKI 3**	14 (0.20%)	19 (1.40%)	

All values presented as mean (95% CI), and categorical variable with the percentage in parentheses. BMI = body mass index, eCCR = estimated creatinine clearance, CPB = cardio-pulmonary bypass, HR = heart rate, SBP = systolic blood pressure, DBP = diastolic blood pressure, CVP = central venous pressure, $PaCO_2$ = arterial CO_2 pressure, PaO_2 = arterial oxygen pressure, SaO_2 = oxygen saturation, ICU = intensive care unit, ESR = erythrocyte sedimentation rate, LDH = lactate dehydrogenase, Hb = hemoglobin, ALAT = alanine aminotransferase, ASAT = aspartate aminotransferase, AKI = acute kidney injury.

Tables of p-values of the DeLong test for difference in areas under the curve for prediction performance of the different Super Learners.

Table 2. Comparison between SL1 and SL2 to SL6

SL1	AUROC [95% CI]	SL2-6	AUROC	Difference
Aortic (SL1)	**0.838 [0.813-0.864]**	Aortic (SL2)	0.825 [0.798-0.852]	0.013
Aortic + CABG (SL1)	**0.799 [0.763-0.835]**	Aortic + CABG (SL3)	0.798 [0.760-0.836]	0.001
Mitral (SL1)	**0.846 [0.812-0.880]**	Mitral (SL4)	0.834 [0.797-0.871]	0.012
Mitral + CABG (SL1)	**0.796 [0.746-0.847]**	Mitral + CABG (SL5)	0.778 [0.723-0.833]	0.018
CABG (SL1)	**0.784 [0.764-0.804]**	CABG (SL6)	0.778 [0.758-0.797]	0.006
All (SL1)	**0.810 [0.798-0.823]**	-	-	-

* denotes statistical significance (p-value < 0.01).

Table 3. Comparison between SL1 and GLM trained with the full cohort per operation type

SL1	AUROC [95% CI]	GLM	AUROC	Difference
Aortic	**0.838 [0.813-0.864]**	Aortic	0.734 [0.655-0.813]	0.104*
Aortic + CABG	**0.799 [0.763-0.835]**	Aortic + CABG	0.690 [0.598-0.782]	0.109*
Mitral	**0.846 [0.812-0.880]**	Mitral	0.810 [0.737-0.883]	0.036
Mitral + CABG	**0.796 [0.746-0.847]**	Mitral + CABG	0.685 [0.550-0.821]	0.111
CABG	**0.784 [0.764-0.804]**	CABG (SL6)	0.750 [0.705-0.796]	0.034
All	**0.810 [0.798-0.823]**	All	0.756 [0.725-0.787]	0.054*

* denotes statistical significance (p-value < 0.01).

Table 4. Comparison between SL2-6 and GLM trained with operation-specific cohorts

SL	AUROC [95% CI]	GLM	AUROC	Difference
Aortic (SL2)	0.825 [0.798-0.852]	Aortic	0.795 [0.727-0.863]	0.030
Aortic + CABG (SL3)	0.798 [0.760-0.836]	Aortic + CABG	0.589 [0.479-0.700]	0.209*
Mitral (SL4)	0.834 [0.797-0.871]	Mitral	0.689 [0.586-0.790]	0.145*
Mitral + CABG (SL5)	0.778 [0.723-0.833]	Mitral + CABG	0.739 [0.610-0.868]	0.039
CABG (SL6)	0.778 [0.758-0.797]	CABG	0.758 [0.739-0.777]	0.020

* denotes statistical significance (p-value < 0.01).

Calibration plots and ECI coefficients for all models.

Table 5. Estimated calibration index (ECI) per model

Model 1	ECI	Model 2	ECI	Difference
Aortic (SL1)	0.291	Aortic (SL2)	**0.199**	0.092
Aortic + CABG (SL1)	**0.249**	Aortic + CABG (SL3)	0.292	-0.043
Mitral (SL1)	0.253	Mitral (SL4)	**0.168**	0.085
Mitral + CABG (SL1)	0.522	Mitral + CABG (SL5)	**0.337**	0.185
CABG (SL1)	**0.067**	CABG (SL6)	0.073	-0.006
All (SL1)	0.149	All (GLM)	**0.084**	0.065

ECI or estimated calibration index is a measure of model calibration developed by van Hoorde et al. (2015). Unlike mean calibration measures which measure calibration at group level, the ECI does it at individual level. The lower the ECI, the better the calibration.

Figure 1. Calibration plot aortic valve (calculated from SL1)

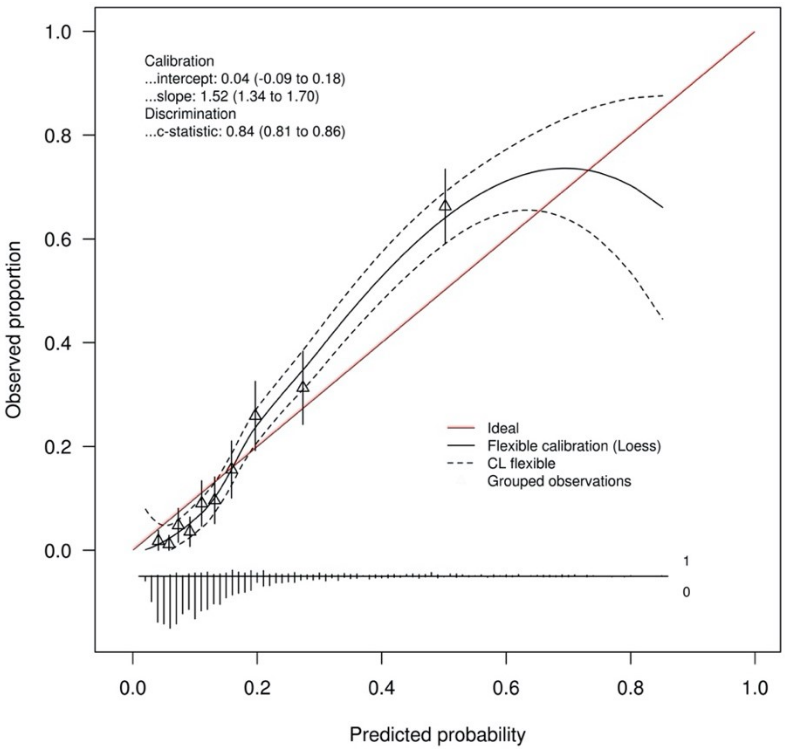

Figure 2. Calibration plot aortic valve (calculated from SL2)

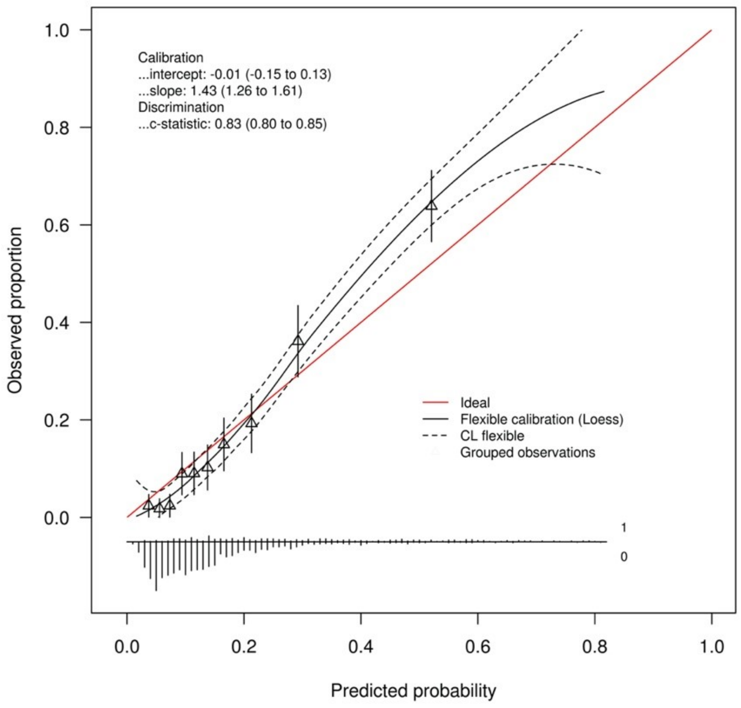

Figure 3. Calibration plot combined aortic valve and CABG (calculated from SL1)

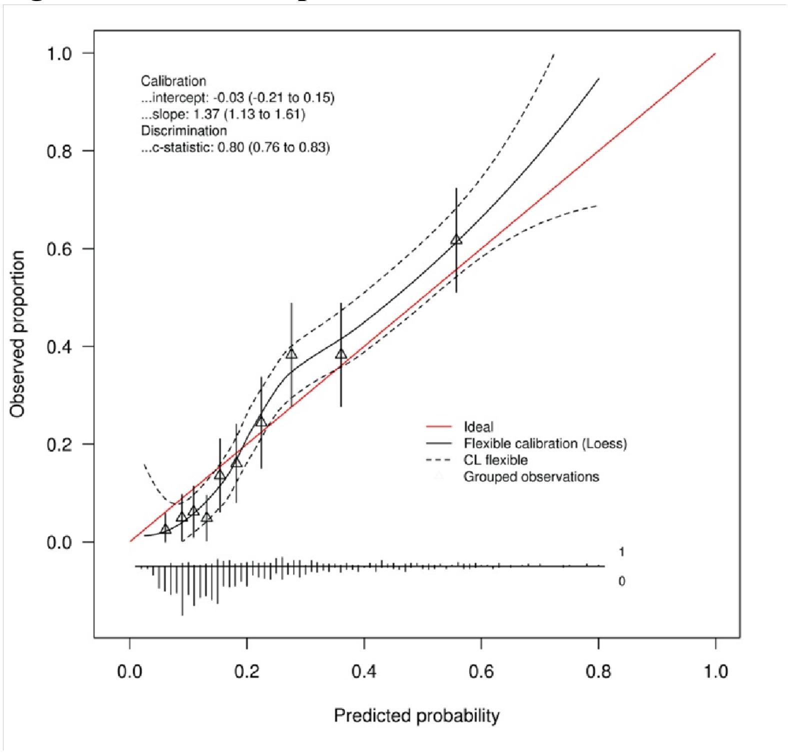

Figure 4. Calibration plot combined aortic valve and CABG (calculated from SL3)

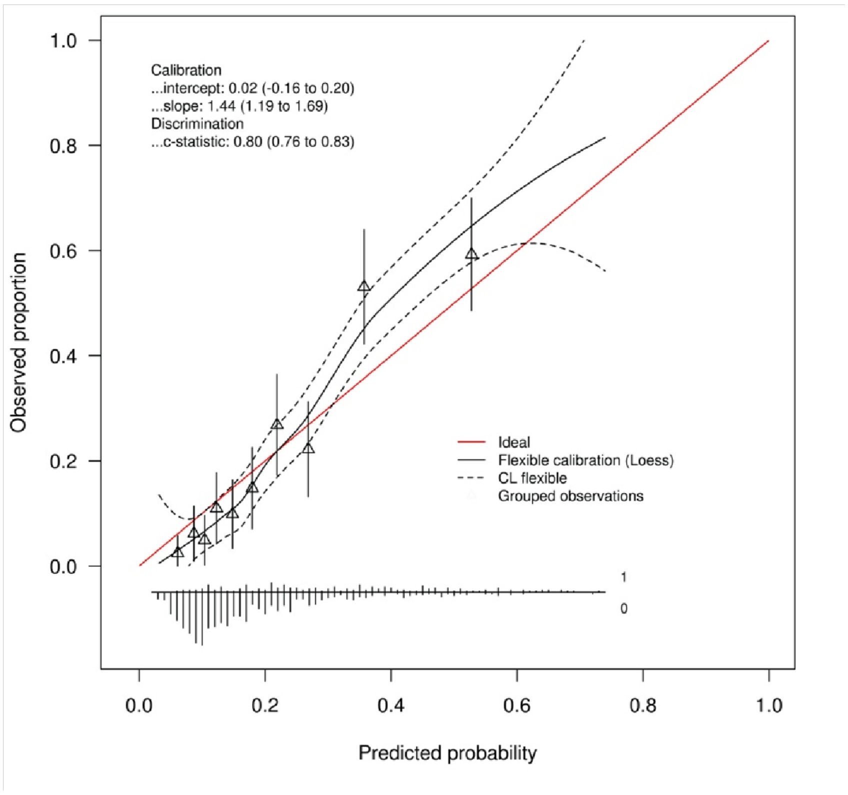

Figure 5. Calibration plot mitral valve (calculated from SL1)

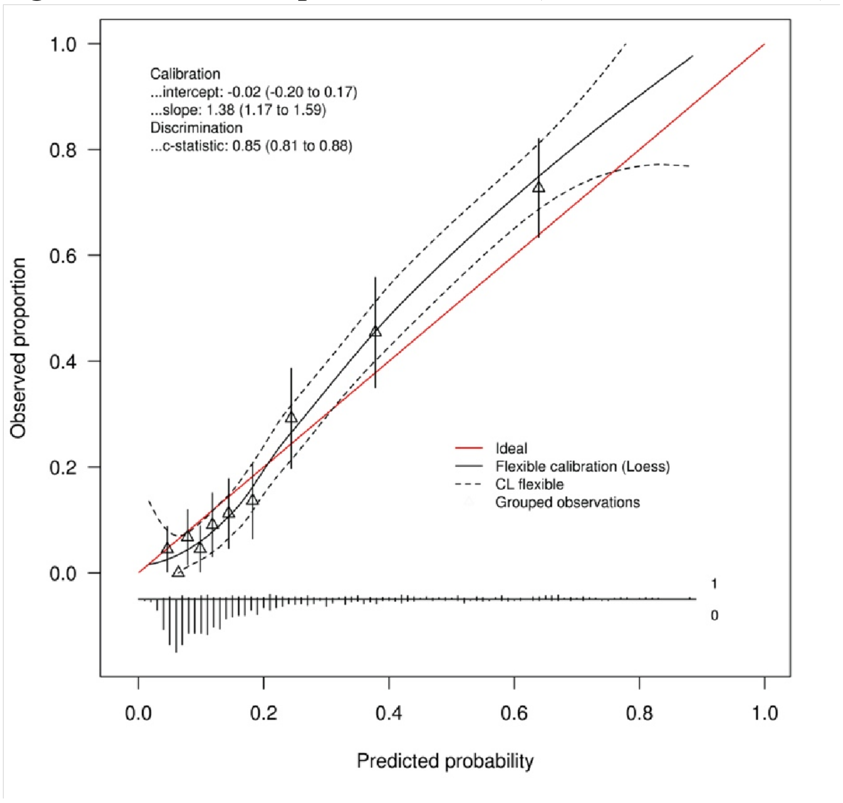

Figure 6. Calibration plot mitral valve (calculated from SL4)

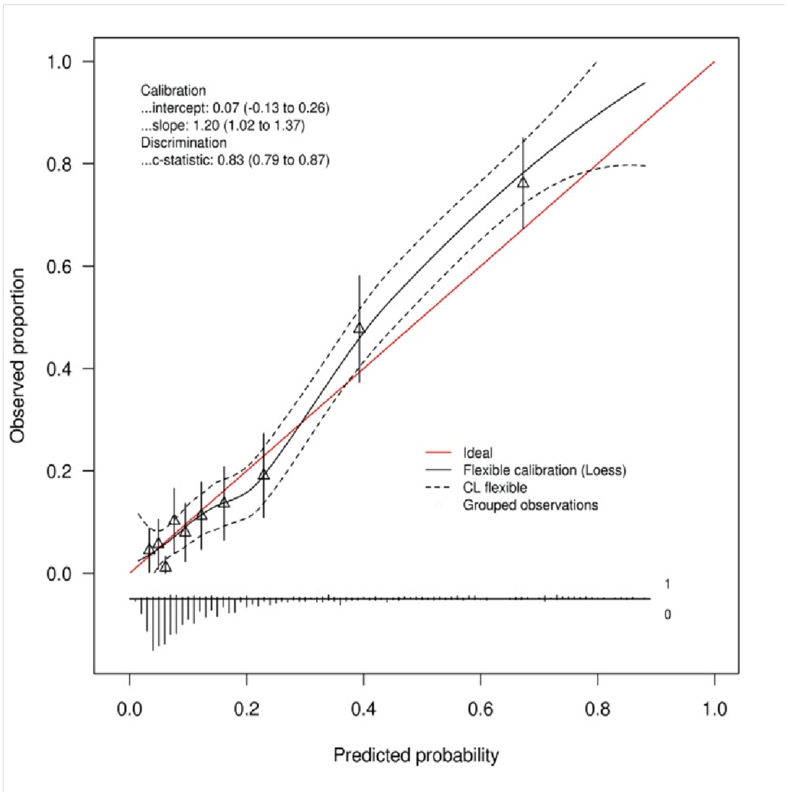

Figure 7. Calibration plot combined mitral valve and CABG (calculated from SL1)

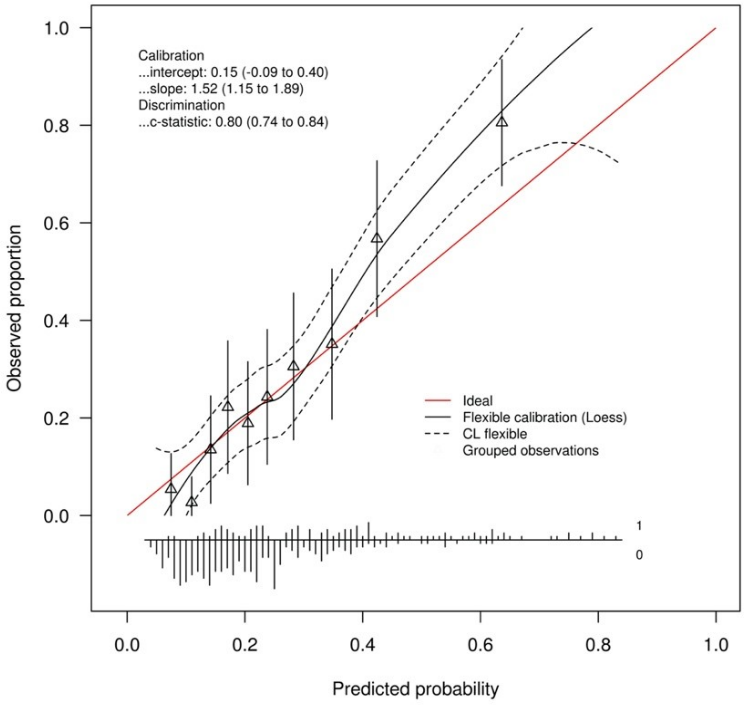

Figure 8. Calibration plot combined mitral valve and CABG (calculated from SL5)

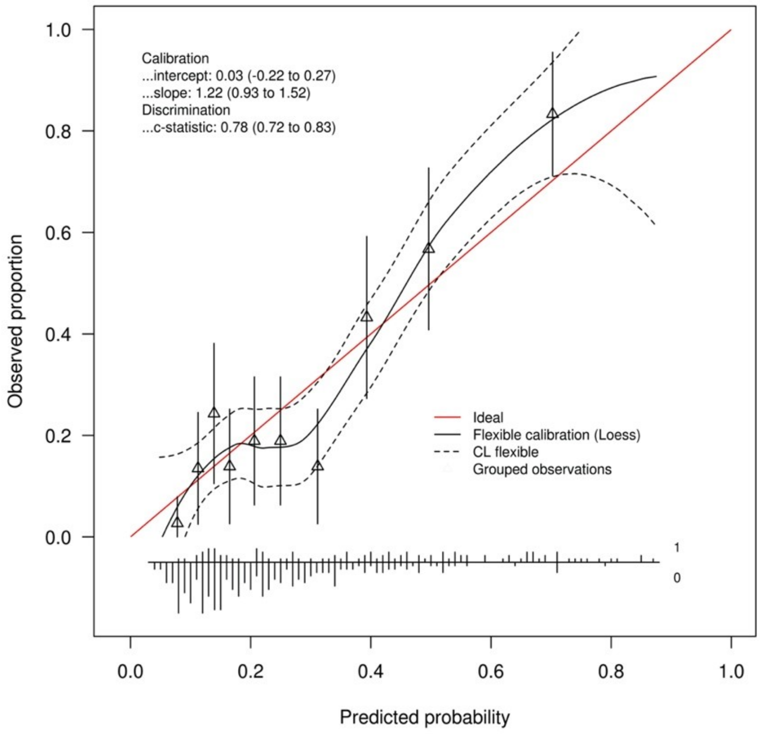

Figure 9. Calibration plot CABG (calculated from SL1)

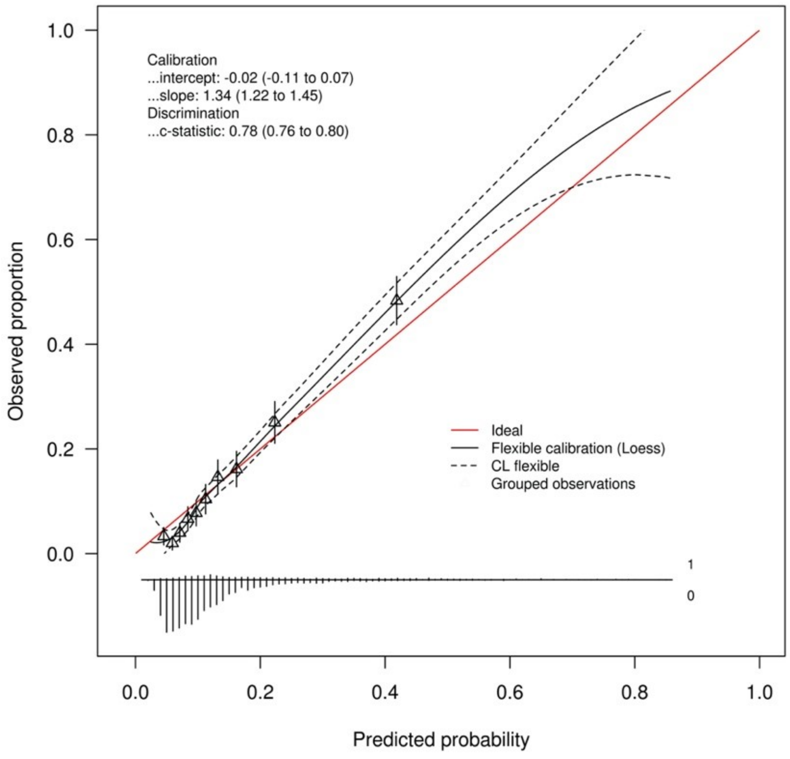

Figure 10. Calibration plot CABG (calculated from SL6)

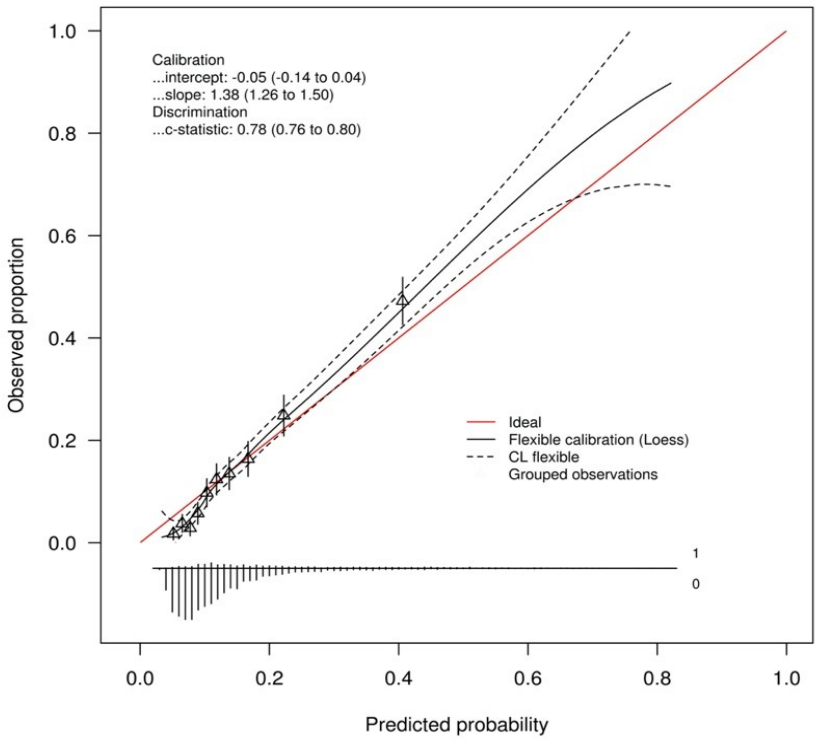

Figure 11. Calibration plot all operations combined (calculated from SL1)

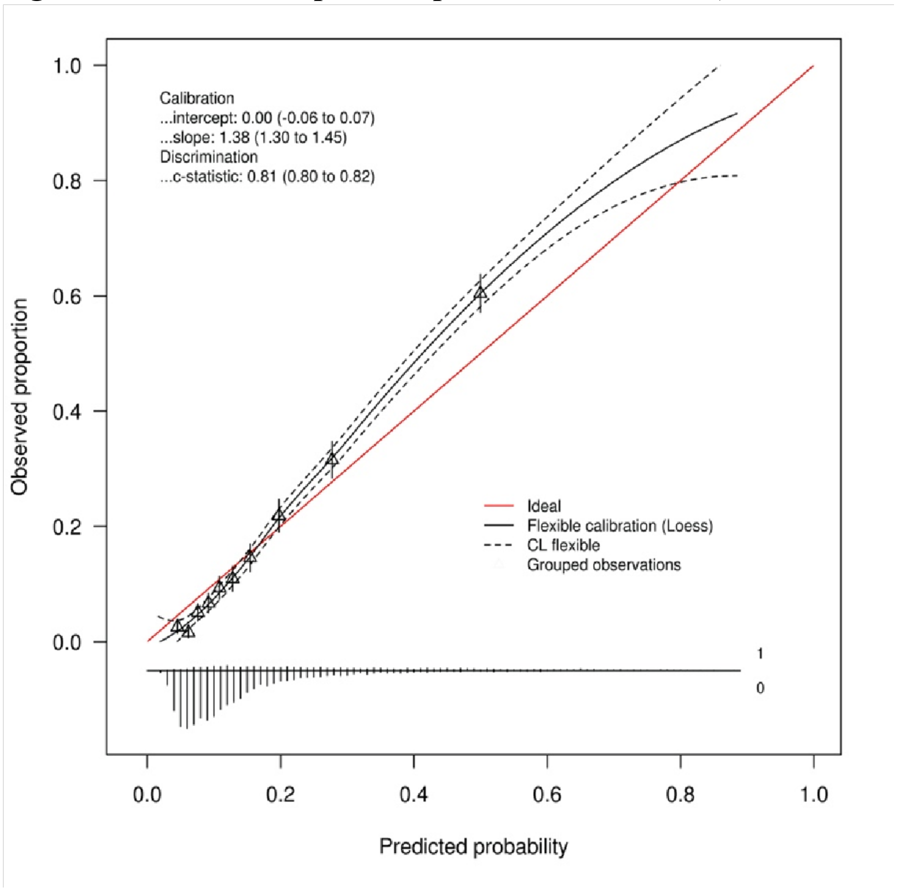

Super Learner confusion matrices (tables 3A-3H).

Table 6
Confusion matrix of the predictions of the Super Learner ensemble trained with aortic valve operations data (n=1663)

	Reference (actual patient outcomes)	
**Prediction with cut-off of 0.50**	Survivors	Non-survivors
Survivors	1363	219
Non-survivors	19	62
**Cut-off at 50% increased risk (0.26)**	Survivors	Non-survivors
Survivors	1227	121
Non-survivors	155	160
**Cut-off with maximum Youden index**	Survivors	Non-survivors
Survivors	1067	77
Non-survivors	315	204

Table 7. Confusion matrix of the predictions of the Super Learner ensemble trained with combined aortic valve and CABG operations data (n=813)

	Reference (actual patient outcomes)	
**Prediction with cut-off of 0.50**	Survivors	Non-survivors
Survivors	627	143
Non-survivors	15	28
**Cut-off at 50% increased risk (0.32)**	Survivors	Non-survivors
Survivors	467	42
Non-survivors	175	129
**Cut-off with maximum Youden index**	Survivors	Non-survivors
Survivors	462	41
Non-survivors	180	130

Table 8. Confusion matrix of the predictions of the Super Learner ensemble trained with mitral valve operations data (n=884)

	Reference (actual patient outcomes)	
**Prediction with cut-off of 0.50**	Survivors	Non-survivors
Survivors	688	104
Non-survivors	22	70
**Cut-off at 50% increased risk (0.30)**	Survivors	Non-survivors
Survivors	644	65
Non-survivors	66	109
**Cut-off with maximum Youden index**	Survivors	Non-survivors
Survivors	639	60
Non-survivors	71	114

Table 9. Confusion matrix of the predictions of the Super Learner ensemble trained with combined mitral valve and CABG operations data (n=367)

	Reference (actual patient outcomes)	
**Prediction with cut-off of 0.50**	Survivors	Non-survivors
Survivors	248	65
Non-survivors	13	41
**Cut-off at 50% increased risk (0.435)**	Survivors	Non-survivors
Survivors	237	53
Non-survivors	24	53
**Cut-off with maximum Youden index**	Survivors	Non-survivors
Survivors	224	41
Non-survivors	37	65

Table 10. Confusion matrix of the predictions of the Super Learner ensemble trained with CABG-only operations data (n=4514)

	Reference (actual patient outcomes)	
**Prediction with cut-off of 0.50**	Survivors	Non-survivors
Survivors	3866	567
Non-survivors	24	57
**Cut-off at 50% increased risk (0.21)**	Survivors	Non-survivors
Survivors	3446	337
Non-survivors	444	287
**Cut-off with maximum Youden index**	Survivors	Non-survivors
Survivors	2752	188
Non-survivors	1138	436

Table 11. Confusion matrix of the predictions of the Super Learner ensemble trained with whole cohort data (n=8241) for each operation subgroup with a cut off of 0.50

**Cut-off of 0.50**	Reference (actual patient outcomes)	
**Aortic valve**	Survivors	Non-survivors
Survivors	1365	230
Non-survivors	17	51
**Aortic valve + CABG**	Survivors	Non-survivors
Survivors	623	137
Non-survivors	19	34
**CABG**	Survivors	Non-survivors
Survivors	3859	559
Non-survivors	31	65
**Mitral valve**	Survivors	Non-survivors
Survivors	687	114
Non-survivors	23	60

Mitral valve + CABG	Survivors	Non-survivors
Survivors	253	76
Non-survivors	8	30
**All**	Survivors	Non-survivors
Survivors	6787	1116
Non-survivors	98	240

Table 12. Confusion matrix of the predictions of the Super Learner ensemble trained with whole cohort data (n=8241) for each operation subgroup with a cut off defined by a 50% increase in mortality risk

**Cut-off at 50% increased risk**	**Reference (actual patient outcomes)**	
**Aortic valve**	Survivors	Non-survivors
Survivors	1251	132
Non-survivors	131	149
**Aortic valve + CABG**	Survivors	Non-survivors
Survivors	570	97
Non-survivors	72	74
**Mitral valve**	Survivors	Non-survivors
Survivors	637	70
Non-survivors	73	104
**Mitral valve + CABG**	Survivors	Non-survivors
Survivors	249	69
Non-survivors	12	37
**CABG**	Survivors	Non-survivors
Survivors	3460	325
Non-survivors	430	299
**All**	Survivors	Non-survivors
Survivors	6152	656
Non-survivors	733	700

Table 13. Confusion matrix of the predictions of the Super Learner ensemble trained with whole cohort data (n=8241) for each operation subgroup with a cut off defined by the maximum Youden index

**Cut-off with maximum Youden index**	**Reference (actual patient outcomes)**	
**Aortic valve (0.159)**	Survivors	Non-survivors
Survivors	1026	57
Non-survivors	356	224
**Aortic valve + CABG (0.207)**	Survivors	Non-survivors
Survivors	462	40
Non-survivors	180	131
**Mitral valve (0.212)**	Survivors	Non-survivors
Survivors	579	44
Non-survivors	131	130
**Mitral valve + CABG (0.274)**	Survivors	Non-survivors
Survivors	203	32

Non-survivors	58	74
**CABG (0.155)**	Survivors	Non-survivors
Survivors	3089	235
Non-survivors	801	389
**All (0.155)**	Survivors	Non-survivors
Survivors	5024	343
Non-survivors	1861	1013

MV = Mitral valve, MV+CABG = combined mitral valve and CABG, AVR = aortic valve, AVR+CA = combined aortic valve and CABG.

Specificity and sensitivity per operation type with adjusted risk thresholds

Table 14. Specificity and sensitivity for the outcome "Non-survivors" for the Super Learners trained on individual operation groups using the default cut-off of 0.50, the cut-off defined by a 50% increase in mortality risk, and the cut-off defined by the maximum Youden index.

	**Default**		**50% increased risk**		**Maximum Youden**	
	Specificity	Sensitivity	Specificity	Sensitivity	Specificity	Sensitivity
AVR	98.6	22.1	88.8	56.9	77.5	72.6
AVR + CABG	97.7	16.4	72.7	75.4	72.7	76.0
MV	96.9	40.2	90.7	62.6	90.0	65.5
MV + CABG	95.0	38.7	90.8	50.0	85.8	61.3
CABG	99.4	9.1	88.9	46.0	70.1	69.9

MV = Mitral valve, MV+CABG = combined mitral valve and CABG, AVR = aortic valve, AVR+CA = combined aortic valve and CABG

Table 15. Specificity and sensitivity for the outcome "Non-survivors" with the Super Learner and GLM models trained on the full cohort using the default cut-off of 0.50, the cut-off defined by a 50% increase in mortality risk, and the cut-off defined by the maximum Youden index.

	**Default**		**50% increased risk**		**Maximum Youden**	
	Specificity	Sensitivity	Specificity	Sensitivity	Specificity	Sensitivity
AVR	98.8	18.2	90.5	53.0	74.3	79.7
AVR + CABG	97.0	19.9	88.8	43.4	72.1	76.6
MV	96.8	34.5	89.7	59.8	81.8	74.7
MV + CABG	96.9	28.3	95.4	34.9	77.8	69.8
CABG	99.2	10.4	89.0	47.9	79.7	62.3
All	98.6	17.7	89.4	51.6	73.3	74.6
GLM	97.2	16.2	85.9	46.8	69.9	70.1

MV = Mitral valve, MV+CABG = combined mitral valve and CABG, AVR = aortic valve, AVR+CA = combined aortic valve and CABG, GLM = generalized linear model.

Peri-operative and pre-operative model comparison

For the comparison between a model built only with pre-operative variables and the full peri-operative model, the following variables were selected: age, gender, body mass index (BMI), pre-operative creatinine, pre-operative urea, pre-operative creatinine clearance (eCCR), pre-operative erythrocyte sedimentation rate (ESR), pre-operative alanine aminotransferase (ALAT), pre-operative aspartate aminotransferase (ASAT), pre-operative lactate dehydrogenase (LDH), pre-operative hemoglobin, pre-operative thrombocytes, and pre-operative leukocytes.

Figure 12. Plot of the Receiver Operating Characteristic (ROC) curves and the respective areas under curve (AUCs) for the Super Learner 1 model using all peri-operative data for the whole cohort and the XGBoost model using only pre-operative data for the whole cohort. SL = Super Learner.

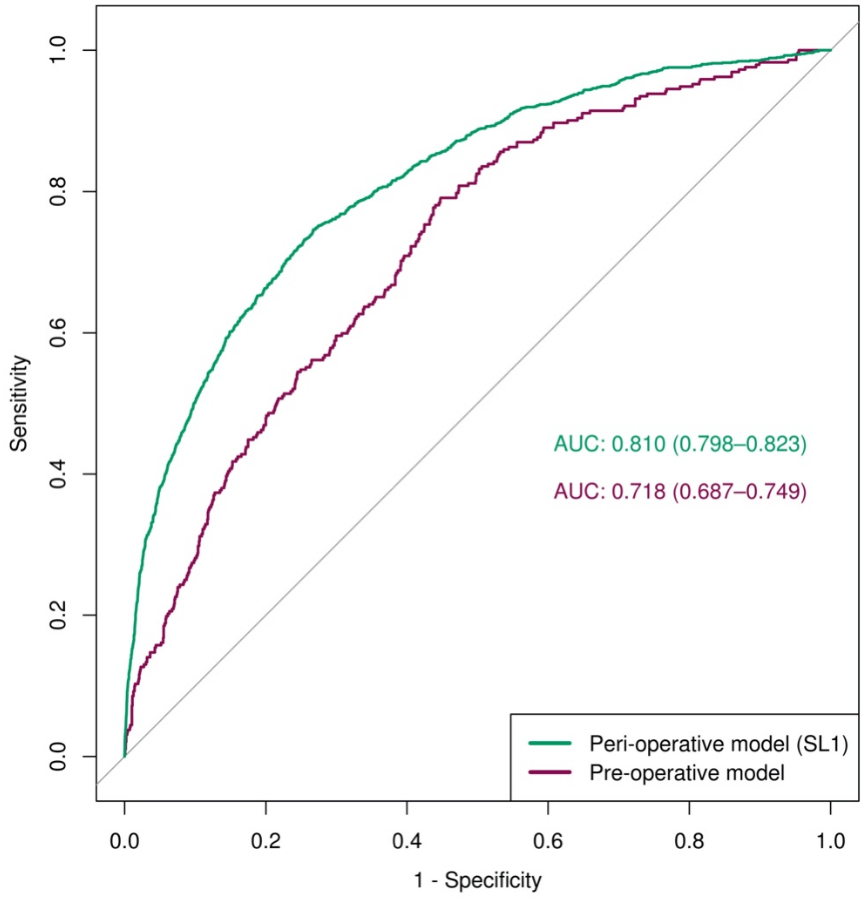

P-value for difference < 0.01

Detailed explanation of the Super Learner, and model hyper-parameter definitions and tuning

The Super Learner algorithm was designed by Dudoit and van der Laan, and is a generalization of the stacking algorithms developed by Breiman (1996), which chooses the optimal regression algorithm from a set of candidates based on a loss function after k-fold-cross-validation (Dudoit and van der Laan, 2006, Legrand et al., 2013). In this process, the dataset is divided into k mutually exclusive and exhaustive subsets of nearly equal size, with one of the k sets serving as a validation set, while the other sets are used for training of each candidate algorithm (Van der Laan, Polley, and Hubbard, 2007). At patient level, this means that each patient is used exactly once in the validation set, and included in the training set for all other rounds

(Pirracchio et al., 2015). For each candidate learner, k risks are calculated and averaged into a "cross-validated risk", based on which the learners with the minimal risk are selected and applied to the entire dataset. These are then included in the new, weighted estimator (the SL), that attributes a relative coefficient to each of the learners that constitute it, so that only those which reduce the calculated risk the most end up contributing to the final weighted prediction. By automatically estimating the weights of the ensemble, and also automatically removing models that do not contribute to the prediction, the SL eliminates the manual tuning and experimentation that a non-automated majority vote or weighed ensemble requires. Moreover, the SL presents individual patient predicted probabilities for 5-year mortality per ensemble and per learner (Polley et al., 2018). This is necessary to define cut-offs for binary classification that increase the relevance and potential clinical applicability of the findings of predictive modelling.

Five candidate algorithms were included in the Super Learner: support bayesian additive regression trees (BART), extremely randomized trees, elastic net, support vector machine (SVM), and extreme gradient boosted machine (XGBoost). To optimize the performance of the algorithm, multiple hyper-parameter combinations were generated for each candidate algorithm. The hyper-parameters tuned and the values defined as optimal are found below, per algorithm.

Support bayesian additive regression trees (BART)

BART is an ensemble-of-trees method which differ from Random Forests and stochastic gradient boosting like Gradient Boosting Machines, in that it relies on an underlying Bayesian probability model, instead of a pure algorithm (Chipman et al., 2010; Kapelner and Bleich, 2016; Breiman, 2001). It sets a number of priors for the structure and the leaf parameters of the trees it generates, which provides additional regularization to the model, and allows for variable importance exploration, including permutation tests and interaction detection. Furthermore, the bartMachine R implementation of this algorithm includes an external predict function that allows forecasts to be generated without the need to re-fit the whole model (Kapelner and Bleich, 2016).

The user-defined hyper-parameters that generate different model configurations are alpha (α), beta (β), and k. The first two, α and β, represent respectively the base and power hyperparameter in the prior probability that a node at a certain depth is nonterminal, which is given by the expression:

$$\alpha(1 + d)^{-\beta}, \alpha \in (0, 1), \beta \in [0,\infty)$$

Chipman et al. (2010) advise taking $\alpha = 0.95$ and $\beta = 2$, respectively, as pre-defined values for these parameters. The higher the alpha, the more splits are encouraged even in situations where predictive gains are modest, which is in line with the tendency of BART to include spurious splits (Chipman et al., 2010). In turn, k determines the prior probability that $E(Y|X)$ is between (-3; 3), which is the number of standard deviations towards each side of the mean. The larger k, the broader the coverage of variances of the provided response values in the training set, and the more conservative the model fit. Seen as the prior specifications for variable selection via BART are a topic of on-going research, we opted for not defining hyper-parameter values for this algorithm, using instead the default α, β, and k.

Extremely randomized trees

This Random Forest-based algorithm available in the R package “extraTrees” is a better performing update to the original “randomForest” R package developed by Liaw and Wiener (2002), mainly due to its novel node splitting process (Simm et al., 2014; Liaw and

Wiener, 2002). It also facilitates parallel processing by building a large number of binary decision trees, independently of each other, and has the ability to learn non-linear models even in large datasets with over 100000 training samples. This algorithm is an extension of Multi-task learning (MTL) to a binary decision tree-based ensemble method, allowing the user to dedicate tree branches to certain specific tasks. This limits the reciprocal influence of multiple tasks on each other, but also reduces a model's ability to capture relevant information from other tasks. The implementation of extraTrees in the Super Learner is rich in hyper-parameterization, especially in sub-task definition. Since we do not define any a priori tasks due to our naïve approach to the data analysis process, data from all tasks is pooled together and no ideal taskwise split hyper- parameter must be optimized. Therefore, only the parameters ntree, which stands for the number of trees to be built, and mtry, which represents the number of features tried at each node, were chosen for tuning. The number of random cuts for each chosen feature was defined as 1, according to the official extraTrees method. Ultimately, 21 extraTree models were used, with mtry ranging from 1 to 7, and ntree as 500, 1000, and 2000.

Elastic net

The biglasso wrapper in the Super Learner implements more computationally-efficient sparse linear and logistic regression models with lasso, ridge, and elastic net penalties by providing a better feature screening process to identify and discard inactive features from the lasso optimization (Zeng and Breheny, 2017). The elastic net algorithm, developed by Zou and Hastie, outperforms lasso-only, as it groups strongly correlated predictors instead of doing covariate selection and is applicable with the "biglasso" package (by adjusting the hyperparameter penalty) (Zou and Hastie, 2005). This method of variable selection, while best applied to cases where the number of predictors (p) is much bigger than the number of observations (n), which is not the case in our dataset, increases the interpretability of the model, a key issue when addressing its possible clinical applicability.

We defined 5 different models, all with a sequential strong rule screening algorithm, but with different mixing values (α) for the elastic net penalty (0.05, 0.4, 0.5, 0.6, and 0.95), which moves from closer to ridge at 0, to close to lasso, at 1, as defined by

$$\alpha\|\beta\|_{\$}+(1-\alpha)/2\|\beta\|^{-}_{-}$$

For lambda, a hyper-parameter which represents the shrinkage penalty for the fitting process and directly affects variance, a maximum of 100 values was tried out by the Super Learner algorithm for optimization (Zeng and Breheny, 2017).

SVM

A Support Vector Machine (SVM) is a class of supervised learning algorithms which classifies data points into two different classes by taking datapoints in a multidimensional space and constructing the hyperplane that best differentiates between the two (Chapelle and Zien, 2005). The projection of the input data to a higher-dimensional space can further be potentiated by the use of kernels, which can increase the efficacy of the trained models. The hyper-parameters to be optimized are the cost (C) and sigma (σ). Cost controls the misclassification tolerance, so that the higher the cost, the harder the margin and the smaller the tolerance for misclassification. In turn, sigma defines the smoothing of the Radial Basis Function kernel we chose. Different values of sigma define how much a single training example influences the model, with a higher sigma constraining the model towards linear. We defined 36 different models for SVM with all possible combinations of cost between 2-2 and 221, and sigma between 2-7 and 2-21. Since a SVM tries to maximize the distance between the separating plane and each support vector (the datapoints closest to the hyperplane), the input of attributes with greater numeric ranges can dominate that of those with smaller ranges (Hastie et al., 2009). Therefore, dummy variables were created for all categorical variables and our numeric predictor variables were centred (subtracting the mean) and scaled (dividing by the standard deviation).

XGBoost

The Extreme Gradient Boosting Machine (XGBoost) algorithm is a scalable tree boosting system used widely in data science, and with very interesting, potentially clinically-oriented properties. Like Ridgeway's original GBM, it is based on the consecutive fitting of new models (base-learners) to the training data set in order to provide a more accurate estimate of the outcome variable (Ridgeway, 2012). Decision trees are combined, and increasingly weigh the "difficult to predict" events to a greater degree, from which a cross validation error is estimated, using k-fold cross-validation (Natekin and Knoll, 2013). The novelty in XGBoost is its use of column sampling, borrowed from Random Forests, and a more solid approach to data sparsity patterns (Chen, 2016).

Seen as the adaptability of XGBoost to a certain problem is determined by its configuration, thoroughly testing a wide range of hyper-parameter configurations is paramount (Kennedy, 2017). The hyper-parameter n trees defines the number of trees (iterations) to be generated by the algorithm, while Max depth defines the maximum number of terminal nodes of a tree (Chen et al., 2018). The learning rate, which controls the rate at which the boosting algorithm descends the error surface, is defined by the shrinkage (or eta) hyper-parameter. The lower the shrinkage value, the more overfitting is prevented by making the boosting process more conservative. We defined 24 different models for XGBoost, with all combinations of n trees at 200, 500, and 1000, Max Depth between 1 and 4, and shrinkage 0.01 and 0.1.

Additional references:

Chipman HA, George EI, McCulloch RE: BART: Bayesian Additive Regression Trees. The Annals of Applied Statistics 2010; 4(1): 266–298.

Breiman L: Random forests. Machine Learning 2001; 45(1):5–32.

Kapelner A, Bleich J: bartMachine: Machine Learning with Bayesian Additive Regression Trees. Journal of Statistical Software 2016; 70(4): 1-40.

Chen T, Guestrin C: XGBoost: A Scalable Tree Boosting System. Proceedings of the International Conference on Knowledge Discovery and Data Mining. 2016; 785-794. DOI: 10.1145/2939672.2939785.

Natekin A, Knoll A: Gradient boosting machines, a tutorial. Front Neurorobot 2013; 7:21.

Ridgeway G: Generalized boosted models: a guide to the gbm package, 2012. URL: https://pdfs.semanticscholar.org/a3f6/d964ac323b87d2de3434b23444cb774a216e.pdf

Simm J, de Abril I, Sugiyama M: Tree-Based Ensemble Multi-Task Learning Method for Classification and Regression. IEICE Trans. Inf. & Syst 2014; 97(6): 1677-1681.

Liaw A, Wiener M: Classification and regression by randomforest. R News, 2(3):18–22, 2002.

Zeng Y, Breheny P: biglasso: Extending Lasso Model Fitting to Big Data. 2017. https://CRAN.Rproject.org/package=biglasso.

Zou H, Hastie T: Regularization and variable selection via the elastic net. Journal of the Royal Statistical Society: Series B (Statistical Methodology) 2005; 67(2): 301-320.
